# Neuromuscular and Kinematic Strategies During Step-Up and Down-Forwards Task in Individuals with Knee Osteoarthritis

**DOI:** 10.3390/jcm15031278

**Published:** 2026-02-05

**Authors:** Denise-Teodora Nistor, Maggie Brown, Mohammad Al-Amri

**Affiliations:** 1 School of Healthcare Sciences, Cardiff University, Heath Park Campus, Cardiff CF14 4XN, UKmaggiebrown3@hotmail.com (M.B.); 2 Biomedical Engineering Department, The Hashemite University, Zarqa 13133, Jordan

**Keywords:** knee osteoarthritis, stepping-up, electromyograph, step ascent, kinematics, stepping-down, step descent

## Abstract

**Background/Objectives:** Knee osteoarthritis (KOA) is associated with pain, functional decline, and altered biomechanics. The Step-Up and Down-Forwards (StUD-F) task provides an ecologically relevant assessment of challenging movements. This study investigated neuromuscular activation and lower-limb kinematics of leading and trailing-limbs during the StUD-F in individuals with KOA. **Methods:** Forty participants with KOA (65.3 ± 7.68 years; 21M/19F; BMI 28.9 ± 4.52 kg/m^2^) completed a 25 cm box StUD-F task. Surface electromyograph recorded bilateral activation of the vastus medialis (VM), vastus lateralis (VL), bicep femoris (BF), and semitendinosus (ST). Triplanar hip, knee, and ankle joint angles were estimated using inertial measurement units. StUD-F events (initial stance; step contact; ascent completion; descent preparation; step-down touchdown; and descent completion) were identified using custom algorithms. Pain was assessed using visual analogue scales and the Knee Injury and Osteoarthritis Outcome Score (KOOS). Limb differences were analysed for leading or trailing roles using paired samples *t*-tests or non-parametric equivalents; waveforms were visually inspected. **Results:** Distinct neuromuscular and kinematic asymmetries were observed when affected and contralateral limbs were compared within each role (leading/trailing). During step-up, the affected leading limb demonstrated higher quadriceps activation at initial stance (VM: *p* = 0.035; VL: *p* = 0.027) and reduced trailing-limb activation at step contact (VM: *p* = 0.015; VL: *p* = 0.018), with sagittal-plane ankle differences (*p* = 0.004). During step-down, when the affected limb initiated ascent, trailing limb activation was higher at descent completion (VL: *p* < 0.001; VM: *p* = 0.003; BF: *p* = 0.009), with coronal-plane hip deviations (*p* < 0.001). When the contralateral limb-initiated ascent, trailing-limb muscles activation differences (VM: *p* < 0.001; VL: *p* = 0.015; BF: *p* = 0.007) and ankle/coronal-plane asymmetries (*p* ≤ 0.049) persisted. **Conclusions:** The StUD-F task elicits altered strategies in KOA, including elevated quadriceps–hamstring co-activation and altered sagittal/coronal alignment, and habitual limb choice across ascent and descent. These adaptations may enhance stability and joint protection but could increase medial compartment loading. The findings support rehabilitation focused on dynamic control, alignment, and shock absorption.

## 1. Introduction

Knee osteoarthritis (KOA) is a progressive degenerative condition involving the articular cartilage, subchondral bone, periarticular musculature, and supporting soft tissues [1,2]. It accounts for approximately 54% of all arthritis cases, with 350,000 adults being diagnosed every year in the UK [3], imposing remarkable economic and social costs [4]. Symptoms such as pain, swelling, and stiffness contribute to functional decline and reduced quality of life [5]. As a leading global cause of disability [6], and with limited therapeutic options [7], understanding the functional implications of KOA remains a critical research priority.

One of the commonly reported functional challenges in KOA is difficulty negotiating steps [8]. These movements are fundamental to activities of daily living (ADL); however, they place considerable mechanical demand on the knee. For this reason, standardised performance tests have been developed to evaluate related functional impairment and inform rehabilitation strategies. The Osteoarthritis Research Society (OARSI) recommends tools such as the ‘Stair Climb Test (SCT)’ and ‘Step-Up-and-Down (StUD)’ test to assess lower limb strength, endurance, balance, and compensatory strategies [9,10]. The Step-Up-and-Over (SUO) task has also been employed to assess functional capacity following knee rehabilitation [11,12,13]. These tools collectively provide a structured measure of performance; however, they vary in the specific biomechanical and neuromuscular demands they impose.

The biomechanical complexity of step and stair negotiation provides further rationale for detailed movement analysis. Step ascent and descent require significant knee flexion and joint loading, which can worsen pain and limit function. While a degree of left–right asymmetry during stair negotiation is observed even among healthy individuals, research indicates that these asymmetries are substantially more pronounced in those with KOA [14,15]. During stair negotiation, individuals with KOA show increased quadriceps recruitment and selective lateral co-activation, which serve to stiffen the knee and support frontal-plane stability [16,17,18]. Quadriceps activation is often delayed, accompanied by reduced sagittal knee excursion and compensatory trunk and hip flexion that redistribute load but compromise movement efficiency [10]. Descent transitions are particularly demanding, with higher fear-avoidance behaviour associated with greater medial co-contraction [14,19]. In turn, these adaptations increase medial compartment loading and may accelerate progression of medial KOA, the most common phenotype [20,21].

While the existing functional assessments capture performance outcomes, they often do not fully characterise the subtle neuromuscular strategies and compensatory kinematic patterns used during natural ADLs [22,23]. To address this limitation, elements of the SUO and StUD tasks were combined to develop a novel functional assessment: the Step-Up and Down-Forwards (StUD-F) task. This task was designed to stimulate more ecologically representative movement behaviours, enabling the examination of dynamically challenging transitions that reflect everyday stair negotiation.

Building on these considerations, the present study investigates neuromuscular activation and lower-limb kinematics during the StUD-F task in individuals with KOA. By analysing both leading and trailing limbs, the study captures the distinct mechanical and neuromuscular demands of ascent and descent. For participants with unilateral KOA, the affected limb was compared to the contralateral limb; in bilateral cases, the more symptomatic limb was designated as affected. With unilateral KOA cases showing an increased likelihood of bilateral structural damage and biomechanical abnormalities [24,25,26], the comparisons between affected and contralateral sides aim to provide insight into relative functional adaptations, rather than comparison to absolute healthy mechanics. This approach enables the precise identification of altered strategies, joint-specific kinematic alterations, and muscle activation differences, thereby advancing understanding of KOA-related functional limitations and informing physiotherapy approaches for stair negotiation.

## 2. Materials and Methods

### 2.1. Participants

Forty adults with clinically diagnosed KOA based on NICE [27] criteria were recruited from the outpatient physiotherapy waiting list and musculoskeletal clinics within Cardiff and Vale University Health Board, Wales, UK. Participants exhibited mild-to-moderate functional severity. Relevant eligibility details are provided in Table 1 [23].

Ethical approval was obtained from the Wales Research Ethics Committee 3 (REC Reference: 23/WA/0311), and all participants provided informed consent prior to enrolment.

### 2.2. Data Collection

#### 2.2.1. Self-Reported Pain

Participants completed the Knee Injury and Osteoarthritis Outcome Score (KOOS) [28] to document self-reported symptoms, pain, functional limitations, sports and recreation function, and knee-related quality of life. Prior to the exercise protocol, participants rated their current knee pain using a 10 cm visual analogue scale (VAS), where 0 represented “no pain” and 10 represented “worst imaginable pain”.

#### 2.2.2. Motion Capture and Electromyography

Lower-limb kinematics were recorded using the Xsens MVN Awinda inertial motion capture system (Movella, Amsterdam, the Netherlands), comprising 17 MTw2 inertial measurement unit (IMU) sensors arranged in a full-body configuration. Joint angles were computed using MVN Analyze Pro software (version 2024.2). Surface electromyography (EMG) was collected bilaterally from the vastus medialis (VM), vastus lateralis (VL), biceps femoris (BF), and semitendinosus (ST) using Delsys Avanti sensors (sampling rate: 2148 Hz). Electrode placement followed SENIAM guidelines [29] and was verified using palpation during isometric contractions. Skin preparation included shaving and sterilising to optimise signal quality. Participants performed isometric maximum voluntary contractions (MVCs) for EMG normalisation. Ground reaction forces (GRFs) were recorded using two force plates (BTS Bioengineering P6000, sampling rate: 1000 Hz) to detect key events.

**Figure 1:**
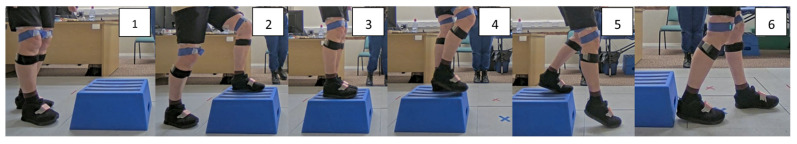
Step-Up and Down-Forwards cycle key events: event 1 = initial stance; event 2 = step contact; event 3 = ascent completion; event 4 = descent preparation; event 5 = step-down touchdown; event 6 = descent completion.

Kinematic and EMG data were simultaneously recorded within the Xsens MVN Analyze Pro version 2024.2.0 software, while GFR data were synchronised through an external trigger generated by the MVN system to ensure precise temporal alignment across all signals. After sensor and electrode placement, participants performed up to eight continuous repetitions of the StUD-F task using a 25 cm box, stepping up with the affected limb leading, followed by eight repetitions with the contralateral limb leading. Further setup details can be found in Supplementary Materials Section S1. Six key events defined each movement cycle (illustrated in Figure 1):

1. Initial stance: last knee extension before step-up initiation.
2. Step contact: initial foot contact on the box.
3. Ascent completion: full foot contact of the trailing limb on the box.
4. Descent preparation: lift off the step-down leading foot.
5. Step-down touchdown: initial ground contact of the step-down leading foot
6. Descent completion: initial ground contact of the step-down trailing foot.

Participants were instructed which leg to use for stepping up to ensure balanced exposure and controlled comparisons. In contrast, participants were free to select the limb for the forward step-down. This design reflects real-world behaviour, allowing freedom in step-down enhanced ecological validity, and aligns with evidence that voluntary limb choice is influenced by effort- and risk-based strategies in goal-directed stepping tasks [14,30]. Previous work also demonstrates that stepping biomechanics depend on both task direction and the chosen lead limb [31], supporting the relevance of capturing self-selected descent patterns. The selected muscles were chosen due to their functional role in mediolateral knee loading: VM and ST contribute to medial knee compartment loading, while the VL and BF influence lateral loading [20,32].

### 2.3. Data Processing and Analysis

All data were processed using MATLAB (version R2023a, MathWorks Inc., Natick, MA, USA). Kinematic data were low-pass filtered using a Butterworth filter with a cut-off frequency of 10 Hz. EMG signals were bandpass filtered (30 Hz–350 Hz), rectified and smoothed with a 150 ms moving average window, and resampled to match the kinematic sampling rate. EMG amplitudes were normalised to each participant’s MVC and expressed as a percentage of MVC. GRF data were likewise resampled to the kinematic sampling frequency. On average, five out of the eight trials per participant met data quality criteria and were included in the analysis.

Task events (1–6) were then identified using custom MATLAB (version R2023a, MathWorks Inc., Natick, MA, USA) algorithms, with event definitions provided in the Supplementary Materials Section S5.1. Pseudocode for the event-detection routines is available in the Supplementary Materials Section S5.2.

### 2.4. Statistical Analysis

Statistical analyses were performed in MATLAB (version R2023a, MathWorks Inc., Natick, MA, USA). Descriptive statistics are reported as means ± standard deviations, and medians (interquartile ranges). Normality was assessed before inferential testing. Differences between affected and contralateral leading or trailing limbs were evaluated using paired-samples *t*-tests or Wilcoxon signed-rank tests during step-up. For step-down, the mixed design comprising paired and unpaired observations necessitated the use of Mann–Whitney U tests to compare affected and contralateral limbs, given their suitability for unequal sample sizes and non-normal distributions. We acknowledge that this test assumes independence, which is partially violated in paired cases; however, this approach enabled the inclusion of all available data. Consequently, statistical inferences should be interpreted with caution. Statistical significance was set at *p* < 0.05.

## 3. Results

### 3.1. Participant Characteristics

Participant characteristics are summarised in Table 2. The cohort included 40 individuals with KOA (65.3 ± 7 years; 21 males, 19 females) and a mean BMI of 28.9 ± 4.5 kg/m^2^. Most participants had unilateral KOA (*n* = 24), with the right limb more commonly affected (*n* = 25) within the participants. No significant differences were found between the affected and contralateral MVCs in any muscle.

### 3.2. Step-Up

Of the 40 participants enrolled in the study, 1 was excluded from paired step-up analysis due to incomplete bilateral data, resulting in *n* = 39 for kinematic comparisons. Additionally, EMG data from 3 participants were excluded because of technical faults, leading to *n* = 36 for EMG analyses.

**Figure 2:**
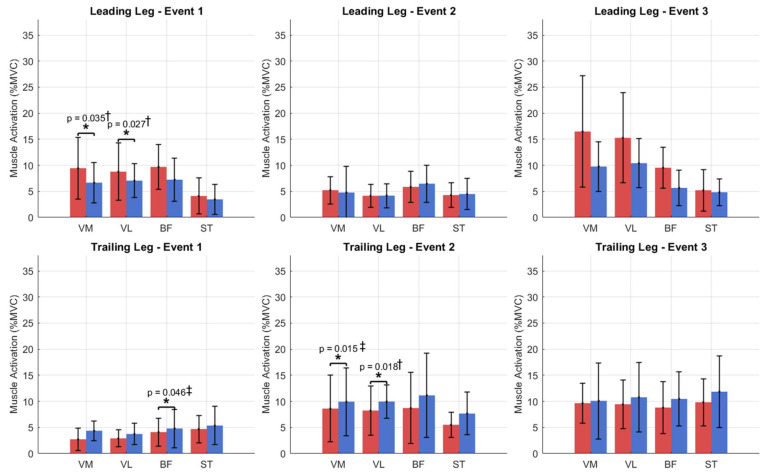
Comparison of muscle activation patterns for quadriceps (VM, VL) and hamstrings (BF, ST) in leading and trailing limbs between affected (red) and contralateral (blue) sides during step-up events (Event 1: initial stance; Event 2: box contact; Event 3: ascent completion). Significant differences (*p* < 0.05) were observed between affected and contralateral limbs across Events 1–3 (* = significant difference). Effect sizes: ‡ = small-to-medium; † = medium effect size.

During step-up, the affected trailing limb exhibited reduced quadriceps activation at Events 1 and 2. In contrast, at Event 3, the affected leading limb demonstrated increased quadriceps activation relative to the contralateral limb, with VM and VL being higher by 7.1% and 2.1%, respectively (Figure 2).

**Figure 3:**
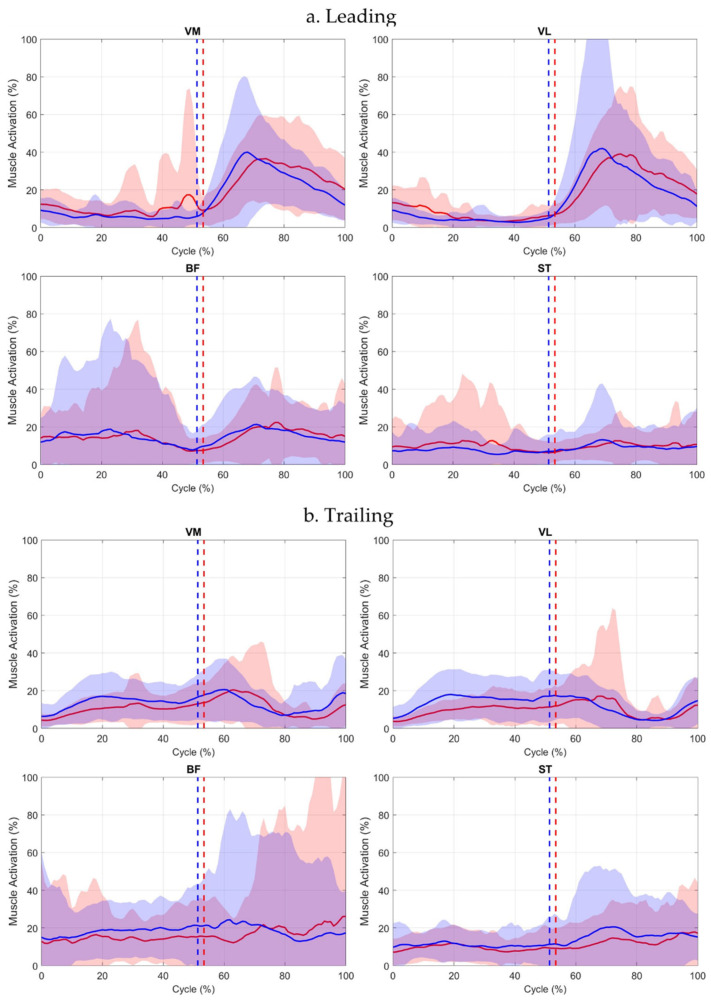
Muscle activation waveforms (mean ± SD) for leading (**a**) and trailing limbs (**b**) across the full step-up cycle. Solid red line = affected limb mean; solid blue line = contralateral limb mean. Red and blue shaded areas represent ±1 SD around the respective mean. Event 1 (initial stance) occurs at 0%. Event 2 (box contact) is indicated by dotted lines (red = affected, blue = contralateral). Event 3 (ascent completion) occurs at 100%.

Waveform inspection indicated notable variability across participants, particularly in BF, and higher VM and ST variability in the affected leading limb between Events 1–2 (Figure 3).

**Figure 4:**
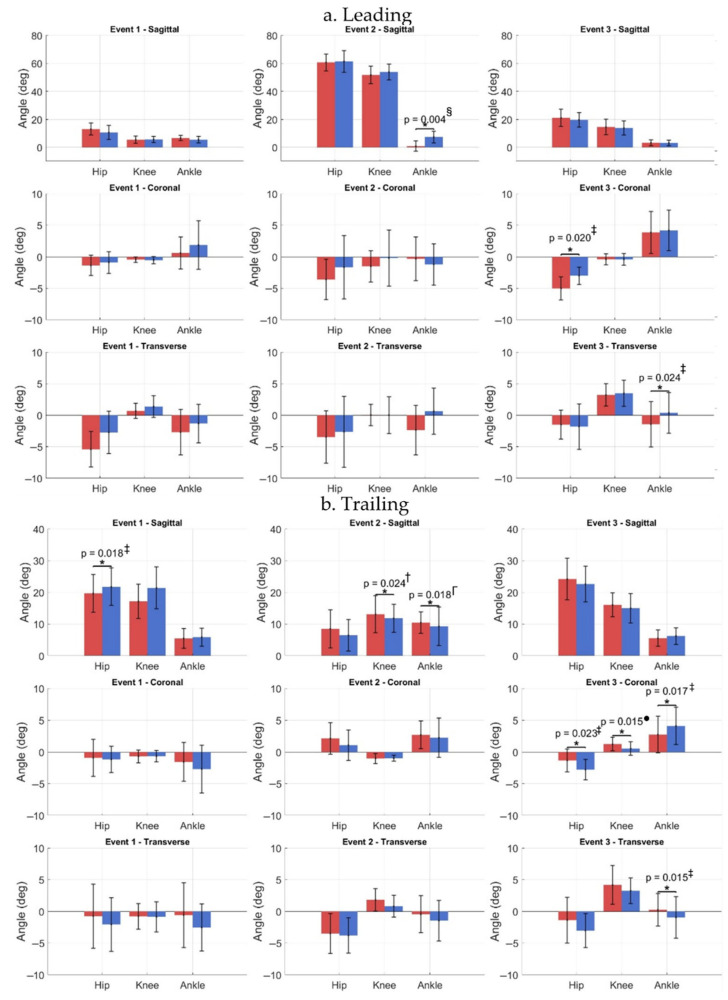
Comparison of hip, knee, and ankle joint angles for leading (**a**) and trailing (**b**) limbs between affected (red) and contralateral (blue) sides during step-up events (Event 1: initial stance; Event 2: box contact; Event 3: ascent completion). Significant differences (*p* < 0.05) were observed between affected and contralateral limbs across Events 1–3 (* = significant difference). Effect sizes: ● = small; ‡ = small-to-medium; † = medium; Γ = medium-to-large; § = large.

**Figure 5:**
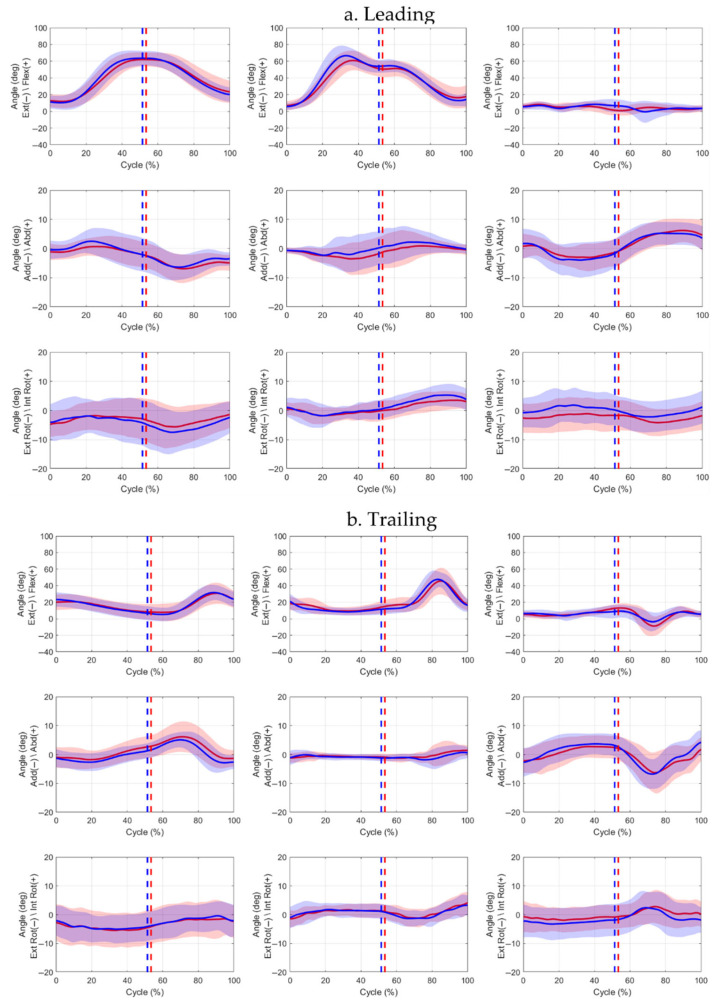
Hip, knee, and ankle joint angle waveforms (mean ± SD) for leading (**a**) and trailing (**b**) limbs across the full step-up cycle. Solid red line = affected limb mean; solid blue line = contralateral limb mean. Red and blue shaded areas represent ±1 SD deviation around the respective mean. Event 1 (initial stance) occurs at 0%. Event 2 (box contact) is indicated by dotted lines (red = affected, blue = contralateral). Event 3 (ascent completion) occurs at 100%.

Kinematic analysis (Figure 4) showed that at Event 1, the leading limb was more extended at the knee and hip than the trailing limb, with increased hip external rotation. Comparison of affected and contralateral limbs revealed that the trailing affected hip was 9.6% less extended At Event 2, the affected leading ankle was less dorsiflexed, whereas the trailing affected knee and ankle were more flexed by 10.7% and 12.5%, respectively. By Event 3, the affected leading hip exhibited greater adduction, and external ankle rotation, while the trailing affected ankle showed internal rotation relative to the contralateral. Waveform inspection indicated reduced sagittal-plane range of motion (ROM) in the affected leading limb and greater transverse-plane variability in contralateral limbs (Figure 5). Further statistics, along with effect sizes, are provided in the Supplementary Materials Section S2.

### 3.3. Step-Down (When Step-Up Leading Leg Was Affected)

Of the 40 participants, 1 was excluded from the kinematic analysis due to an inability to complete the task bilaterally (*n* = 39), and EMG data from three participants were removed because of technical faults, resulting in *n* = 36 for EMG analyses. When participants led step-up with the affected leg, they stepped down with the affected limb in 70% of the trials. EMG analysis values can be found in Table 3.

**Figure 6:**
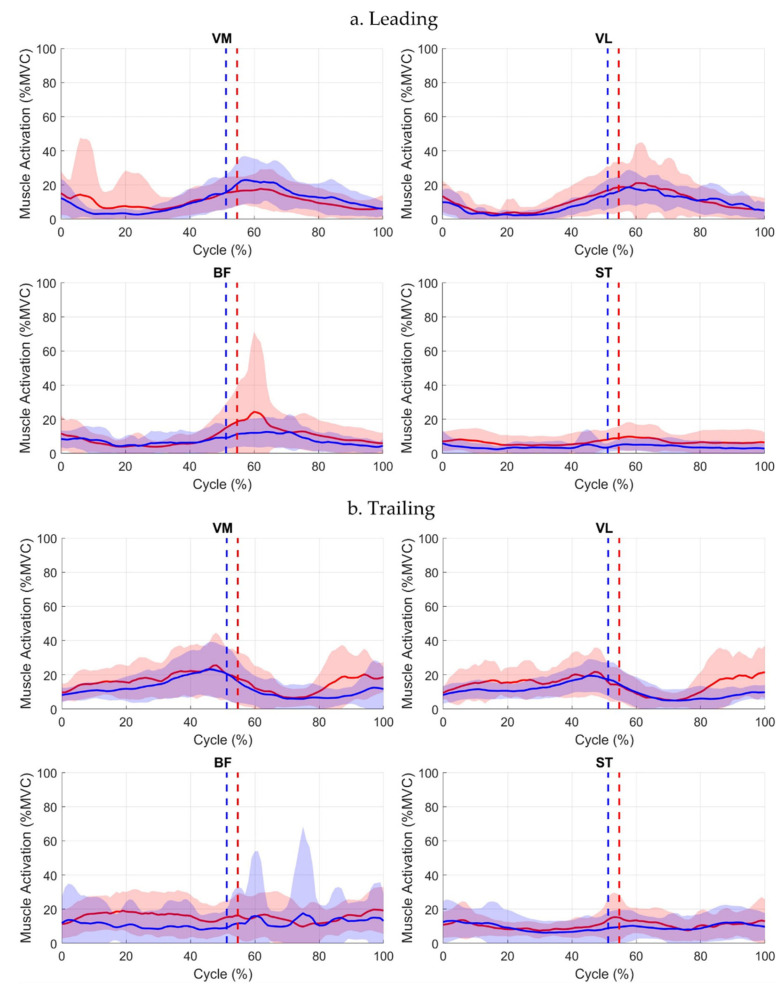
Muscle activation waveforms (mean ± SD) for leading (**a**) and trailing (**b**) limbs across the full step-down (when step-up leading limb was affected) cycle. Solid red line = affected limb mean; solid blue line = contralateral limb mean. Red and blue shaded areas represent ±1 SD deviation around the respective mean. Event 4 (descent initiation) occurs at 0%. Event 5 (leading-leg touchdown) is indicated by dotted lines (red = affected, blue = contralateral). Event 6 (descent completion).

The analysis showed that the leading leg exhibited significantly higher ST activity at Event 6 when compared to the contralateral, whereas the trailing affected leg showed higher BF activation at Event 5 and 6, as well as higher quadriceps activation at Event 6 (Table 3, Figure 6). Further statistics are provided in the Supplementary Materials Section S3.

**Figure 7:**
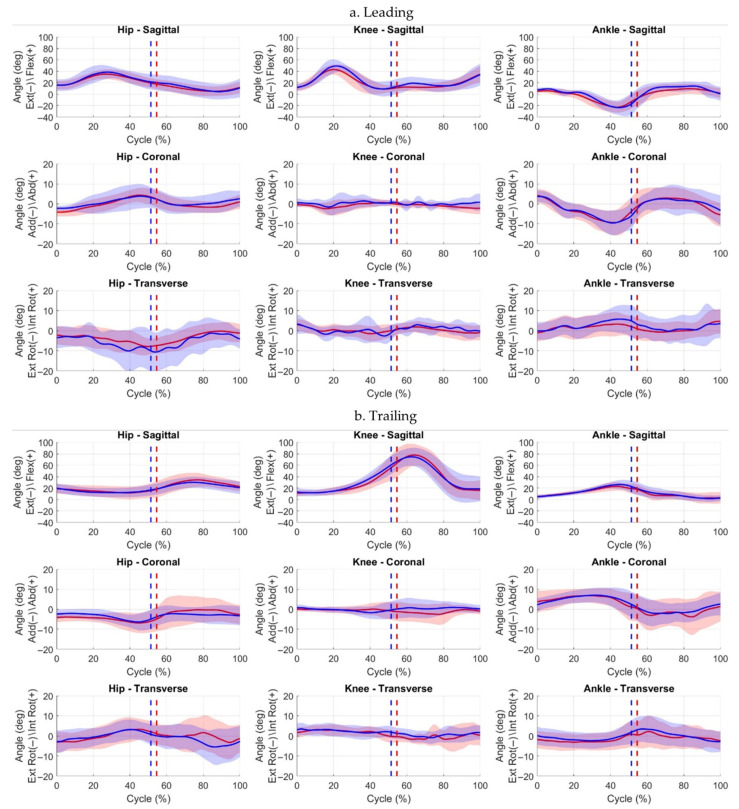
Hip, knee, and ankle joint angle waveforms (mean ± SD) for leading (**a**) and trailing (**b**) limbs across the full step-down (when step-up leading limb was affected) cycle. Solid red line = affected limb mean; solid blue line = contralateral limb mean. Red and blue shaded areas represent ±1 SD deviation around the respective mean. Event 4 (descent initiation) occurs at 0%. Event 5 (leading-leg touchdown) is indicated by dotted lines (red = affected, blue = contralateral). Event 6 (descent completion) occurs at 100%.

Kinematic analysis showed that at Event 4, the affected leading hip was 12.5% significantly less adducted (*p* < 0.001), while the trailing hip was 28.9% more adducted (*p* = 0.012) compared to the contralateral limb. Waveform inspection indicated decreased sagittal-plane ROM in the affected leading limb and reduced variability in most planes and joints (Figure 7).

### 3.4. Step-Down (When Step-Up Leading Leg Was Contralateral)

Of the 40 participants, due to technical faults, 1 was excluded from kinematic analysis (*n* = 39), and EMG data from 3 participants were removed, resulting in *n* = 36 for EMG analyses.

**Figure 8:**
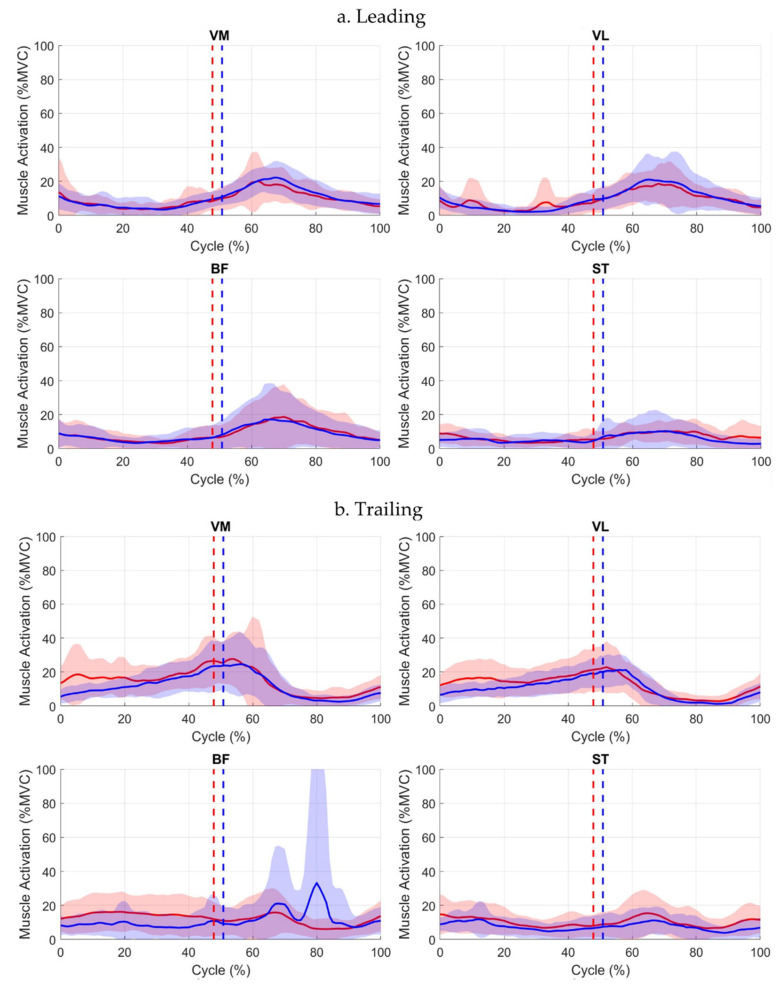
Muscle activation waveforms (mean ± SD) for leading (**a**) and trailing (**b**) limbs across the full step-down (when step-up leading limb was contralateral) cycle. Solid red line = affected limb mean; solid blue line = contralateral limb mean. Red and blue shaded areas represent ±1 SD deviation around the respective mean. Event 4 (descent initiation) occurs at 0%. Event 5 (leading-leg touchdown) is indicated by dotted lines (red = affected, blue = contralateral). Event 6 (descent completion) occurs at 100%.

When the contralateral limb led step-up, the affected limb led step-down in 34.6% of trials. EMG analysis (Table 3) showed significantly higher ST activation in the affected leading leg at Event 4; trailing VM, VL, and BF muscles were also significantly elevated. At Event 5, the trailing affected BF was raised significantly, whereas at Event 6, ST affected activation was higher than the contralateral. Waveform inspection revealed a notable spike in contralateral trailing BF between Events 5–6 (Table 3, Figure 8).

**Figure 9:**
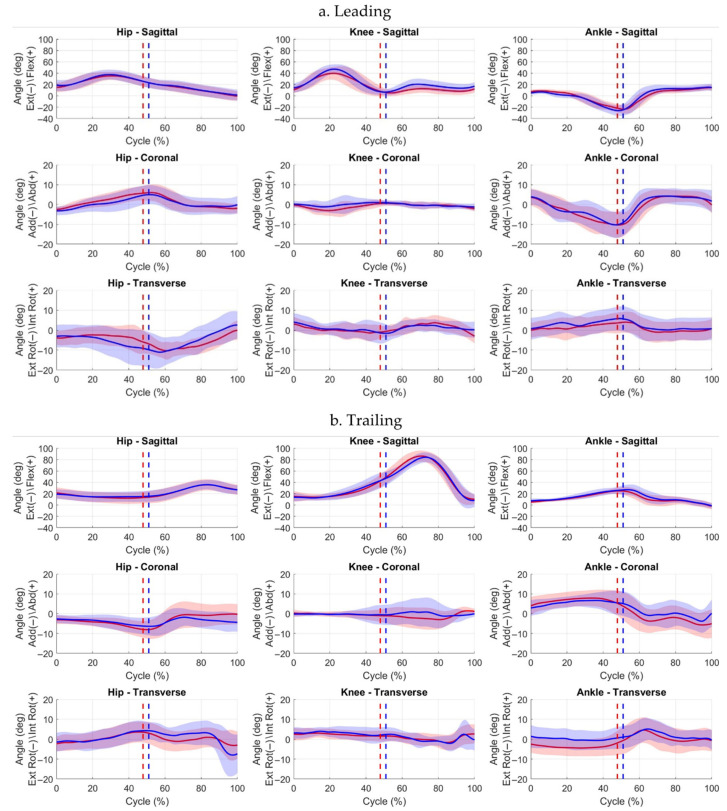
Hip, knee, and ankle joint angle waveforms (mean ± SD) for leading (**a**) and trailing (**b**) limbs across the full step-down (when step-up leading limb was contralateral) cycle. Solid red line = affected limb mean; solid blue line = contralateral limb mean. Red and blue shaded areas represent ±1 SD deviation around the respective mean. Event 4 (descent initiation) occurs at 0%. Event 5 (leading-leg touchdown) is indicated by dotted lines (red = affected, blue = contralateral). Event 6 (descent completion) occurs at 100%.

**Figure 10:**
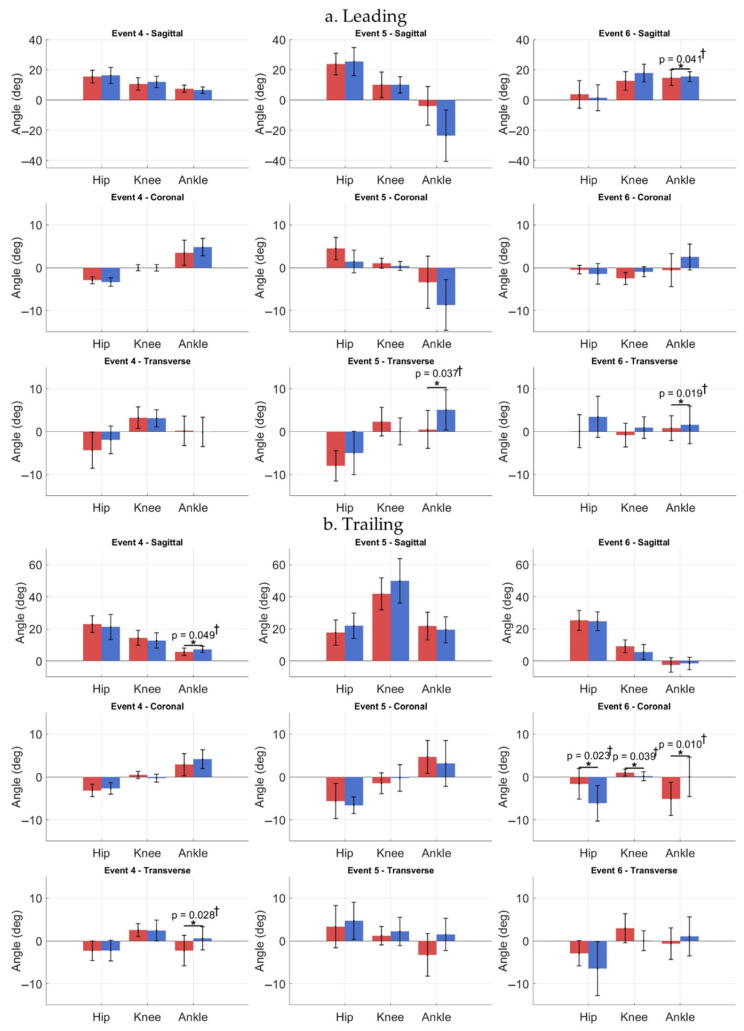
Comparison of hip, knee, and ankle joint angles for leading (**a**) and trailing limbs (**b**) between affected (red) and contralateral (blue) sides during full step-down (when step-up leading limb was contralateral) cycle. Solid red line = affected limb; solid blue line = contralateral limb. Event 4 (descent initiation) occurs at 0%. Event 5 (leading-leg touchdown) is indicated by dotted lines. Significant differences (*p* < 0.05) observed between affected and contralateral limbs across Events 1–3 (* = significant difference). Effect sizes: † = medium.

When comparing affected and contralateral limbs, kinematics showed that at Event 4, the affected trailing ankle was significantly less dorsiflexed and more externally rotated. At Event 5, the leading affected hip was more abducted/externally rotated, with the ankle significantly less externally rotated. The trailing affected limb had reduced knee/hip flexion. At Event 6, the leading affected ankle was less dorsiflexed and internally rotated, and the trailing affected hip, knee, and ankle showed significant coronal-plane differences. Reduced sagittal knee ROM persisted in the affected leading limb, with coronal- and transverse-plane variability in trailing and contralateral limbs (Figure 9 and Figure 10).

Further statistics can be found in Supplementary Materials Section S4.

## 4. Discussion

This study examined the neuromuscular and triplanar kinematics strategies in individuals with KOA during a continuous STuD-F task, examining both trailing and leading limb mechanics. Through analyses of EMG and kinematic patterns across hip, knee, and ankle, we provided new insights into how KOA alters functional movement strategies during a task that closely resembles everyday mobility demands. Overall, participants adopted distinctive altered strategies in the affected limb that appeared to prioritise joint stability and load management rather than pain avoidance. These adaptations were evident through increased quadriceps–hamstring co-activation, altered sagittal and coronal-plane joint alignment, and reliance on habitual limb-use patterns. It is important to note that left–right asymmetries are present in healthy populations. However, in individuals with KOA, these asymmetries are magnified, with significantly larger discrepancies in joint loading, kinetics, and muscle activation [14,15]. This supports the fact that the observed asymmetries are disease-related, not just a natural variability.

During step-up, the affected leading limb exhibited higher quadriceps activation at the initial stance (Event 1), accompanied by the co-contraction of the hamstrings in the trailing limb. This pattern likely represents a stabilisation-focused strategy, consistent with evidence linking quadriceps recruitment to pain protection and knee joint control [33]. Inter-participant variability was particularly evident in BF activity, as well as in medial quadriceps and hamstrings activity, indicating that individuals with KOA dynamically adjust muscle recruitment to maintain stability, albeit with potentially inefficient load distribution [34]. Kinematic findings show that the affected leading limb demonstrated reduced sagittal knee ROM, a more extended knee, and increased hip external rotation, while the trailing limb showed deviations in the coronal plane. These adaptations indicate preparatory adjustments aimed at negotiating the step safely and managing frontal-plane instability.

Between Events 1 and 2, delayed quadriceps activation in propulsion and ongoing hamstring engagement in the trailing limb were observed. These patterns, alongside high waveform variability, particularly in BF, point to altered co-contraction strategies to support knee stability under load. Increased variability in VM, VL, and ST activation in the symptomatic limb aligns with Roberts et al. [35], who reported increased VL excitation during step-up swing in both limbs. However, excessive medial co-contraction could exacerbate medial compartment loading and potentially accelerate the progression of medial KOA, the most common phenotype [20,21]. In contrast, the affected trailing limb activation was comparatively lower during ascent, reflecting limitations in push-off strength. Kinematic data showed reduced sagittal knee ROM in the symptomatic leading limb further supporting the existing evidence that diminished ROM and muscle weakness contribute to altered joint mechanics [35,36]. In contrast, the contralateral limb exhibited greater hip and ankle transverse-plane variability, possibly reflecting greater mobility and adaptability on the less symptomatic side.

During the transition phase onto the box (Event 2 to Event 3), delayed quadriceps activity in the leading limb persisted, contrasting with Hinman et al.’s findings [37], but consistent with Iijima et al.’s [10] systematic review on stair ambulation. Persistent variability in BF and quadriceps activation in the trailing limb suggests sustained lateral stability demands as body weight transferred onto the step. By the end of ascent, the leading limb demonstrated clear quadriceps dominance, reinforcing the use of altered neuromuscular strategies to support propulsion. Kinematically, the affected leading limb exhibited increased hip adduction and ankle external rotation, while the trailing limb demonstrated marked coronal-plane deviations across joints. These features likely represent balance adjustments and rotational asymmetries that may increase fall risk [38].

Allowing participants to freely select their descent limb provided insight into habitual movement strategies. Most participants used the same limb to lead ascent and descent, reflecting a step-by-step pattern typical of KOA stair negotiation. Interestingly, when the contralateral limb-initiated ascent, the affected leg led descent in only 34.6% of trials, leaving the trailing affected limb as the supporting leg in most descents. This finding challenges the assumption that individuals with KOA consistently avoid loading the affected side. Instead, limb selection appeared to be governed by habitual motor patterns and movement continuity rather than pain avoidance. This behaviour has clear biomechanical consequences, consistent with research showing that limb preference influences descent mechanics [14,30,39].

At descent preparation (Event 4), both scenarios demonstrated elevated quadriceps and hamstring activation in the trailing support limb, suggesting increased stabilisation demands in early descent [40,41]. When ascent was initiated with the contralateral limb, the affected leading limb showed the highest ST activity and increased hip external rotation. In contrast, ascent with the affected limb was associated with reduced hip adduction and greater trailing-leg hip flexion, indicating subtle differences in weight-shift strategies. Descent was also characterised by greater hip rotational variability in the contralateral limb, which may reflect adapted mobility. These findings are consistent with previous reports of reduced knee sagittal ROM during stair descent [42] and single-step tasks [35].

At ground contact (Event 5), both conditions showed similar deviations in the affected leading limb, including increased hip abduction and external rotation, and reduced ankle plantarflexion. These findings suggest impaired control during touchdown. Trailing-limb mechanics, however, differed by condition. With contralateral-led ascent, trailing limb, quadriceps, and hamstring activity remained high, accompanied by reduced knee and hip flexion, compromising shock absorption. When ascent was led with the affected limb, trailing-limb activation shifted toward the BF recruitment, highlighting reliance on frontal-plane stabilisation strategies. These strategies align with evidence that KOA reduces knee and hip flexion during descent, typically compensated for increased quadriceps–hamstring co-activation and selective BF recruitment to stabilise the frontal plane [16,17,18].

As participants completed descent (Event 6), asymmetries persisted regardless of which limb initiated ascent. When ascent was initiated with the contralateral limb, the trailing affected limb showed increased medial co-contraction and varus-associated malalignment patterns (reduced hip adduction, increased knee abduction, and greater ankle adduction), which are likely to elevate medial compartment loading [43]. In contrast, ascent initiated with the affected limb produced sustained higher muscle activation throughout descent, reflecting a more rigid, protective strategy as the limb transitioned to full load bearing. This aligns with previous evidence of pain-protective descent strategies and altered stabilisation to maintain joint control in KOA [14,16,44].

Importantly, despite minimal acute pain immediately prior to testing (VAS 1.32 ± 1.69), participants demonstrated moderate chronic symptoms and functional limitations, with substantial difficulties occurring in higher demand and severe impact on overall quality of life based on KOOSs. This suggests that altered strategies were not solely driven by pain, but represents longer-term functional adaptations aimed at maintaining stability and joint protection. Variability in EMG and kinematics across individuals further indicates that KOA induces heterogeneous movement strategies, potentially influenced by symptom severity, habitual patterns, and residual strength asymmetries. This interpretation aligns with previous research showing that neuromuscular alterations in KOA persist even in low-pain states [14,30].

This study is the first to conduct a continuous, triplanar analysis of leading and trailing limbs during a StUD-F task in KOA, allowing participants to self-select the descending limb. The ecological strength of this approach offers insights into real-world step negotiation and stair tasks, moving beyond standardised protocols that may underestimate altered strategies. Findings emphasise the importance of examining both limbs during functional assessments and rehabilitation planning. Interventions should address altered quadriceps and hamstring co-activation, sagittal-plane limitations, and coronal-plane deviations to optimise stability and reduce medial compartment loading.

These findings emphasise the importance of examining both limbs during functional assessment and rehabilitation planning. Targeted, task-specific rehabilitation interventions may help address the observed deficits. For example, isokinetic exercises [45] and biofeedback [46] may optimise quadriceps and hamstring recruitment and reduce inefficient co-contraction. Flexion-extension mobility drills and progressive stair training, potentially supported by inferential electrotherapy [47], may improve sagittal-plane knee ROM, functional mobility, and shock absorption. Frontal-plane deviations, including increased hip adduction and coronal-plane instability, suggest a role for hip abductor strengthening, core stabilisation [48,49], and lateral stepping exercises to enhance dynamic balance and alignment. Finally, the habitual limb-use asymmetries observed highlight the potential benefit of bilateral-loading symmetry training, such as alternating-limb stair negotiation and functional balance exercises, to mitigate medial compartment loading. Together, these interventions address specific neuromuscular and mechanical deficits associated with KOA, promoting safer, more efficient movement and potentially slowing disease progression.

There are, however, some limitations to consider. Structural severity of KOA was not confirmed radiologically within the bounds of the study, although participants had prior clinical diagnoses that may have included imaging. The focus on functional movement justified the use of KOOS to assess severity due to its reliability and responsiveness, although this limits structural characterisation. This still provides valuable insight into the condition’s impact on daily function. Approximately 40% of participants reported bilateral involvement, which may have influenced contralateral limb outcomes, but this aligns with evidence that most individuals with KOA eventually develop bilateral symptoms [24,25,26]. Consequently, contralateral limbs may not represent true controls, and comparisons reflect intra-individual differences rather than healthy reference values. The absence of an asymptomatic control group limits broader generalisation, though the study design prioritised ecological validity within a rehabilitation context. Unequal step-down sample sizes, minor EMG data loss (7.5%), and occasional supra-maximal EMG values (>100% MVC) were inherent to task demands and technical constraints [16]. Temporal misalignment from custom event detection was mitigated through visual verification. Effect-size measures were included alongside *p*-values to enhance biomechanical interpretation.

Despite these limitations, the study provides novel contributions, particularly through continuous, triplanar analysis of both limbs during an ecologically valid stepping task.

## 5. Conclusions

Individuals with KOA exhibit distinctive, task-specific neuromuscular and kinematic adaptations during continuous step-up and step-down movements. These include elevated quadriceps and hamstring co-activation, altered sagittal- and coronal-plane joint alignment, and reliance on habitual limb-use patterns. Such strategies appear to prioritise stability and joint protection, even when pain is relatively low, but may inadvertently increase medial compartment loading and potentially contribute to disease progression. Participants often selected the same limb for both ascent and descent, highlighting the influence of entrenched movement habits on functional task execution.

Overall, by exploring intra-individual adaptations in bilateral KOA, our findings emphasise the need for rehabilitation that is both limb-specific and task-specific, with a focus on targeting dynamic control, joint alignment, and shock absorption to support safer and more efficient movement in daily life. Future research should continue to investigate KOA biomechanics in ecologically valid environments and develop assessment protocols that better represent how individuals negotiate real-world movement tasks.

## Figures and Tables

**Table 1:** Eligibility criteria.

Inclusion Criteria	Exclusion Criteria
Adults aged 45 years or older.	Where the knee is not identified by the participant as the main source of pain, e.g., comorbid painful conditions, widespread pain.
Clinical diagnosis of knee OA.	Contraindication to exercise.
Referred for physiotherapy for clinically diagnosed knee OA.	Pain caused by malignancy, fractures or inflammatory arthritis.
Activity-related joint pain.	Has received surgery for their knee pain in the last 12 months or had previous knee arthroplasty on the affected knee.
Self-reported knee pain on most days for the past 3 months.	Has commenced new treatment for knee pain during the preceding 12-week pre-enrolment.
Average pain severity in the past week of 4 or greater on 10-point Numeric Rating Scale.	
Able to understand written and spoken English.	
Able to provide written informed consent.	

**Table 2:** Participant characteristics.

Age (yrs)			65.3 ± 7.68
Sex (M/F)			21/19
Height (m)			1.70 ± 0.11
Mass (kg)			83.7 ± 15.3
Body mass index (kg/m^2^)			28.9 ± 4.52
KOA type (unilateral/bilateral)			24/16
More affected side (L/R)			15/25
Dominant side (L/R)			7/33
VAS score			1.32 ± 1.69
KOOS Domain Scores	Symptoms		50.5 ± 20.6
	Pain		50.7 ± 15.4
	Functional limitations		61.3 ± 15.6
	Sports		36.3 ± 22.6
	QoL		35.2 ± 15.3
MVC (mV)	VM	Affected	0.13 (0.14)
		Contralateral	0.16 (0.18)
	VL	Affected	0.27 (0.31)
		Contralateral	0.30 (0.28)
	BF	Affected	0.27 (0.23)
		Contralateral	0.21 (0.32)
	ST	Affected	0.33 (0.50)
		Contralateral	0.27 (0.28)

Values represent mean ± standard deviation or median (interquartile ranges). L = left. R = right. KOA = knee osteoarthritis. VAS = visual analogue scale. KOOS = Knee Injury and Osteoarthritis Outcome Score. MVC = maximum voluntary contraction. VM = vastus medialis. VL = vastus lateralis. BF = bicep femoris. ST = semitendinosus.

**Table 3:** Leading- and trailing-leg Mann–Whitney U significance testing for muscle activation (VM, VL, BF, ST) across step-down (when step-up leading leg was affected or contralateral) Events 4–6 (Event 4: descent initiation; Event 5: leading-leg touchdown; Event 6: descent completion).

Leg Role	Event	Affected Leading							Non-Affected Leading					
		Muscle	Affected Median (%MVC)	Affected IQR (%MVC)	Contralateral Median (%MVC)	Contralateral IQR	*p*	r	Affected Median (%MVC)	Affected IQR (%MVC)	Contralateral Median (%MVC)	Contralateral IQR (%MVC)	*p*	r
Leading	Event 4	VM	12.004	11.262	9.898	20.726	0.385	0.12	9.220	10.120	10.306	8.599	0.639	0.07
		VL	12.274	11.261	12.035	15.138	0.374	0.16	6.937	5.720	8.656	7.602	0.374	0.50
		BF	8.560	10.588	9.032	7.136	0.865	0.13	6.341	10.299	5.317	6.029	0.569	0.12
		ST	5.050	6.519	3.187	5.734	0.385	0.20	9.514	7.250	4.006	5.815	0.014	0.34
	Event 5	VM	8.331	11.799	9.883	14.981	0.816	0.02	11.203	14.362	11.335	11.595	0.881	0.08
		VL	7.168	8.934	6.028	10.262	0.539	0.09	7.167	10.513	6.954	10.764	0.757	0.38
		BF	4.191	6.512	4.162	4.995	0.832	0.12	6.750	11.260	4.310	10.443	0.529	0.34
		ST	2.703	4.646	3.740	5.411	0.352	0.02	7.097	7.638	3.411	6.464	0.080	0.24
	Event 6	VM	3.640	4.597	7.619	8.333	0.498	0.03	4.697	7.975	7.621	7.692	0.503	0.02
		VL	2.998	4.218	4.120	5.071	0.582	0.09	2.910	7.491	6.089	6.948	0.303	0.07
		BF	3.133	4.038	2.438	4.870	0.330	0.09	3.972	7.018	3.429	3.368	0.803	0.04
		ST	4.373	5.432	2.054	2.119	0.004	0.04	3.165	6.572	2.299	2.514	0.147	0.02
Trailing	Event 4	VM	9.090	8.232	7.773	6.189	0.253	0.03	14.349	11.140	4.003	3.780	0.000	0.09
		VL	8.131	7.422	7.031	4.598	0.150	0.39	11.363	9.268	6.595	8.615	0.015	0.39
		BF	8.470	8.555	6.756	7.287	0.512	0.13	10.230	9.705	6.659	6.155	0.007	0.24
		ST	5.975	14.125	10.501	11.752	0.899	0.24	10.611	13.374	8.093	4.302	0.084	0.11
	Event 5	VM	15.430	16.397	19.251	13.998	0.539	0.10	17.307	17.246	14.583	22.537	0.610	0.09
		VL	17.812	15.097	15.208	9.793	0.767	0.42	16.250	13.782	18.830	21.068	0.897	0.25
		BF	12.907	14.534	3.993	12.256	0.006	0.08	10.455	9.824	2.471	7.532	0.006	0.14
		ST	8.650	5.767	5.334	6.003	0.086	0.57	4.843	8.918	3.666	7.687	0.418	0.17
	Event 6	VM	17.907	13.325	9.604	8.930	0.003	0.14	10.227	10.822	6.627	6.565	0.071	0.03
		VL	16.363	7.926	9.950	5.082	0.000	0.37	8.642	5.557	8.384	6.878	0.212	0.10
		BF	13.159	14.052	5.689	9.869	0.009	0.40	10.936	12.432	9.787	11.606	0.466	0.20
		ST	10.195	8.516	6.334	11.916	0.472	0.10	9.765	9.764	4.728	2.792	0.027	0.31

MVC = maximum voluntary contraction. IQR = interquartile range. r = biserial-rank correlation. VM = vastus medialis. VL = vastus lateralis. BF = bicep femoris. ST = semitendinosus.

## Data Availability

Analysed data can be found in the Supplementary Materials. Raw data can be accessed by request.

## Supplementary Materials

The following supporting information can be downloaded at https://www.mdpi.com/article/10.3390/jcm15031278/s1, Table S1: Leading-leg descriptive statistics and Wilcoxon significance testing for muscle activation (VM, VL, BF, ST) across step-up events 1–3 (Event 1: initial stance; Event 2: box contact; Event 3: ascent completion); Table S2: Trailing-leg descriptive statistics and Wilcoxon significance testing for muscle activation (VM, VL, BF, ST) across step-up events 1–3 (Event 1: initial stance; Event 2: box contact; Event 3: ascent completion); Table S3: Leading-leg descriptive statistics and paired-samples *t*-test/Wilcoxon significance testing for triplanar (sagittal, coronal, transverse) lower-limb joint angles (hip, knee, ankle) across step-up events 1–3 (Event 1: initial stance; Event 2: box contact; Event 3: ascent completion); Table S4: Trailing-leg descriptive statistics and paired-samples *t*-test/Wilcoxon significance testing for triplanar (sagittal, coronal, transverse) lower-limb joint angles (hip, knee, ankle) across step-up events 1–3 (Event 1: initial stance; Event 2: box contact; Event 3: ascent completion); Table S5: Leading-leg descriptive statistics and Mann–Whitney U significance testing for muscle activation (VM, VL, BF, ST) across step-down (when step-up leading leg was affected) events 4–6 (Event 4: descent initiation; Event 5: leading-leg touchdown; Event 6: descent completion); Table S6: Trailing-leg descriptive statistics and Mann–Whitney U significance testing for muscle activation (VM, VL, BF, ST) across step-down (when step-up leading leg was affected) events 4–6 (Event 4: descent initiation; Event 5: leading-leg touchdown; Event 6: descent completion); Table S7: Leading-leg descriptive statistics and Mann–Whitney U significance testing for triplanar (sagittal, coronal, transverse) lower-limb joint angles (hip, knee, ankle) across step-down (when step-up leading-leg was affected) events 4–6 (Event 4: descent initiation; Event 5: leading leg touchdown; Event 6: descent completion); Table S8: Trailing-leg descriptive statistics and Mann–Whitney U significance testing for triplanar (sagittal, coronal, transverse) lower-limb joint angles (hip, knee, ankle) across step-down (when step-up leading leg was affected) events 4–6 (Event 4: descent initiation; Event 5: leading-leg touchdown; Event 6: descent completion); Table S9: Leading-leg descriptive statistics and Mann–Whitney U significance testing for muscle activation (VM, VL, BF, ST) across step-down (when step-up leading leg was contralateral) events 4–6 (Event 4: descent initiation; Event 5: leading-leg touchdown; Event 6: descent completion); Table S10: Trailing-leg descriptive statistics and Mann–Whitney U significance testing for muscle activation (VM, VL, BF, ST) across step-down (when step-up leading-leg was contralateral) events 4–6 (Event 4: descent initiation; Event 5: leading-leg touchdown; Event 6: descent completion); Table S11: Leading-leg descriptive statistics and Mann–Whitney U significance testing for triplanar (sagittal, coronal, transverse) lower-limb joint angles (hip, knee, ankle) across step-down (when step-up leading-leg was contralateral) events 4–6 (Event 4: descent initiation; Event 5: leading-leg touchdown; Event 6: descent completion); Table S12: Trailing-leg descriptive statistics and Mann–Whitney U significance testing for triplanar (sagittal, coronal, transverse) lower-limb joint angles (hip, knee, ankle) across step-down (when step-up leading-leg was contralateral) events 4–6 (Event 4: descent initiation; Event 5: leading-leg touchdown; Event 6: descent completion); Figure S1: Horizontal and vertical views of Step-Up and Down-Forwards setup with force platforms (FPs), box location, and dimensions; Figure S2: Comparison of hip, knee, and ankle joint angles in leading limbs between affected (red) and contralateral (blue) sides during (a) step-down (when step-up leading limb was affected) events; (b) step-down (when step-up leading limb was contralateral) events. Event 4: descend initiation; Event 5: leading-leg touchdown; Event 6: descent completion. Significant differences (*p* < 0.05) were observed between affected and contralateral limbs across Events 4–6; Figure S3: Comparison of hip, knee, and ankle joint angles in trailing limbs between affected (red) and contralateral (blue) sides during (a) step-down (when step-up leading limb was affected) events; (b) step-down (when step-up leading limb was contralateral) events. Event 4: descent initiation; Event 5: leading leg touchdown; Event 6: descent completion. Significant differences (*p* < 0.05) were observed between affected and contralateral limbs across Events 4–6; Figure S4 Comparison of hip, knee, and ankle joint angles in leading limbs between affected (red) and contralateral (blue) sides during (a) step-down (when step-up leading limb was affected) events; (b) step-down (when step-up leading limb was contralateral) events. Event 4: descent initiation; Event 5: leading-leg touchdown; Event 6: descent completion. Significant differences (*p* < 0.05) were observed between affected and contralateral limbs across Events 4–6; Figure S5: Comparison of hip, knee, and ankle joint angles in trailing limbs between affected (red) and contralateral (blue) sides during a) step-down (when step-up leading limb was affected) events; b) step-down (when step-up leading limb was contralateral) events. Event 4: descent initiation; Event 5: leading-leg touchdown; Event 6: descent completion. Significant differences (*p* < 0.05) were observed between affected and contralateral limbs across Events 4–6.

## Abbreviations

The following abbreviations are used in this manuscript:

KOA	Knee osteoarthritis
StUD-F	Step-Up and Down-Forwards
M	Male
F	Female
BMI	Body mass index
VM	Vastus medialis
VL	Vastus lateralis
BF	Bicep femoris
ST	Semitendinosus
ADL	Activity of daily living
OARSI	Osteoarthritis Research Society
SCT	Stair climb test
StUD	Step-Up-and-Down
SUO	Step-Up-and-Over
UK	United Kingdom
REC	Research Ethics Committee
KOOS	Knee Injury and Osteoarthritis Outcome Score
VAS	Visual analogue scale
GRF	Ground reaction force
EMG	Electromyography
MVC	Maximum voluntary contraction
IMU	Inertial movement unit
SD	Standard deviation
IQR	Interquartile range
ROM	Range of motion
