# Supplementary material for: Neuromuscular and Kinematic Strategies During Step-Up and Down-Forwards Task in Individuals with Knee Osteoarthritis
Source: J Clin Med. 2026 Feb 5;15(3):1278. doi: 10.3390/jcm15031278 (PMC12897695; doi:10.3390/jcm15031278)
Supplement: Supplementary file 1 [file jcm-15-01278-s001.zip › jcm-4073355-supplementary.pdf]

Supplementary Materials

1. ***Step-Up and Down-Forwards Exercise Set-up***

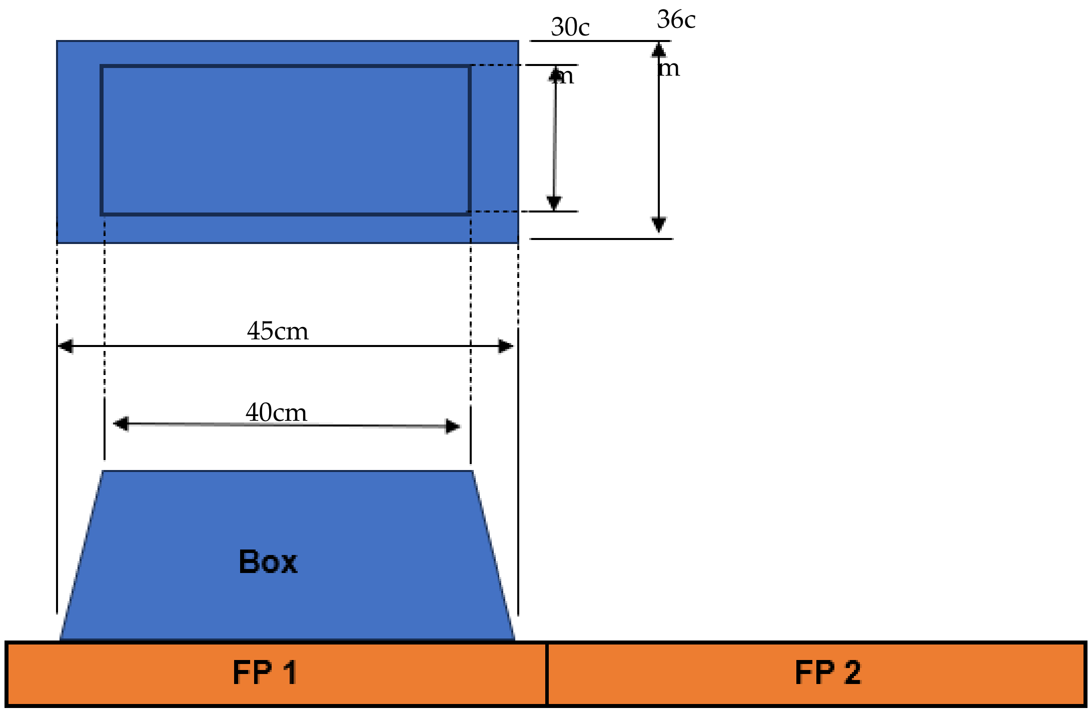

), box location, and dimensions.

2. Step-Up

2.1. EMG

2.1.1 Descriptive Statistics

Table S1. Leading-leg descriptive statistics and Wilcoxon significance testing for muscle activation (VM, VL, BF, ST) across step-up events 1-3 (Event 1: initial stance; Event 2: box contact; Event 3: ascent completion).

Event	Muscle	N	Affected Mean (%MVC)	Affected SD (%MVC)	Affected Median (%MVC)	Affected IQR (%MVC)	Contralateral Mean (%MVC)	Contralateral SD (%MVC)	Contralateral Median (%MVC)	Contralateral IQR (%MVC)	p	r
Event 1	VM	36	11.5870	7.0425	9.4395	11.8953	8.8929	6.2106	6.6639	7.8309	0.035	0.35
	VL	36	12.7959	8.5586	8.7720	10.9963	8.5819	5.4910	7.0565	6.4818	0.027	0.37
	BF	36	12.5958	9.6951	9.6966	8.6639	10.2200	10.2220	7.2444	8.3468	0.064	0.31
	ST	36	6.7033	5.2669	4.1156	6.9659	6.7362	10.4119	3.4606	5.7745	0.116	0.26
Event 2	VM	36	7.5498	7.9159	5.2273	5.2624	9.2816	11.2832	4.7859	10.0326	0.116	0.12
	VL	36	6.3731	5.8991	4.1476	4.4855	7.3109	8.6231	4.1765	4.5928	0.131	0.02
	BF	36	7.4701	5.6121	5.8593	5.9543	8.6790	8.6068	6.4696	7.1211	0.192	0.04
	ST	36	6.5231	6.8498	4.2892	4.6276	6.3165	7.8713	4.4760	5.9424	0.198	0.06
Event 3	VM	36	20.1917	16.8426	16.4923	21.3631	11.6270	7.8839	9.7679	9.5056	0.460	0.41
	VL	36	17.5616	12.5038	15.2798	17.3505	10.4300	5.3181	10.4163	9.4327	0.925	0.40
	BF	36	10.7577	8.7940	9.5340	7.7831	8.3880	7.1229	5.6590	6.8417	0.802	0.29
	ST	36	7.5217	6.1429	5.2108	7.9657	5.5809	3.9400	4.8329	5.1933	0.718	0.25

Table S2. Trailing-leg descriptive statistics and Wilcoxon significance testing for muscle activation (VM, VL, BF, ST) across step-up events 1-3 (Event 1: initial stance; Event 2: box contact; Event 3: ascent completion).

Event	Muscle	N	Affected Mean (%MVC)	Affected SD (%MVC)	Affected Median (%MVC)	Affected IQR (%MVC)	Contralateral Mean (%MVC)	Contralateral SD (%MVC)	Contralateral Median (%MVC)	Contralateral IQR (%MVC)	p	r
Event 1	VM	36	4.1992	3.2530	2.7334	4.3002	6.4733	7.3945	4.3750	3.7885	0.167	0.26
	VL	36	3.5627	2.6543	2.9079	3.2634	5.6414	6.4774	3.7506	4.1472	0.055	0.25
	BF	36	6.0968	4.2474	4.1171	5.3208	7.8422	7.2883	4.8055	7.3303	0.046	0.22
	ST	36	6.2626	5.8423	4.7087	5.2334	7.7379	6.2064	5.3684	7.3600	0.116	0.21
Event 2	VM	36	10.7751	7.8295	8.6262	12.8195	13.0673	8.7858	9.9294	13.0456	0.015	0.23
	VL	36	9.2072	5.7717	8.2422	9.4978	13.0389	10.3939	9.9560	6.3624	0.018	0.32
	BF	36	13.1834	14.6939	8.7246	13.5921	15.8334	13.2343	11.1485	16.2050	0.087	0.33
	ST	36	8.6587	12.0438	5.5181	4.7825	9.5584	6.4767	7.6658	8.1495	0.136	0.26
Event 3	VM	36	11.0519	8.3465	9.6507	7.6187	17.6219	18.4474	10.0961	14.5365	0.157	0.24
	VL	36	10.3325	7.1153	9.4401	9.2713	13.9454	11.4558	10.7789	13.3371	0.460	0.12
	BF	36	12.3311	11.9200	8.8156	9.9091	13.3252	10.0159	10.4664	10.4366	0.128	0.25
	ST	36	12.0506	11.0688	9.8261	9.0450	13.0563	8.5878	11.8591	13.6923	0.338	0.16

2.2. Kinematics

2.2.1 Descriptive Statistics

Table S3. Leading-leg descriptive statistics and paired-samples t-test/Wilcoxon significance testing for triplanar (sagittal, coronal, transverse) lower-limb joint angles (hip, knee, ankle) across step-up events 1-3 (Event 1: initial stance; Event 2: box contact; Event 3: ascent completion).

Event	Joint	Plane	N	Affected Mean (º)	Affected SD (º)	Affected Median (º)	Affected IQR (º)	Contralateral Mean	Contralateral SD (º)	Contralateral Median (º)	Contralateral IQR (º)	p	d/r
**Event 1**	**Hip**	**Sag**	39	12.701	7.668	13.013	8.564	11.219	7.864	10.607	10.134	0.101*	0.17*
		**Cor**	39	-1.142	2.817	-1.384	3.217	-0.352	3.131	-0.904	3.443	0.332*	0.04*
		**Tran**	39	-4.677	4.269	-5.427	5.619	-4.002	6.268	-2.730	6.717	0.530*	0.13*
	**Knee**	**Sag**	39	6.911	5.346	5.454	5.042	5.808	4.136	5.645	4.345	0.302ɣ	0.27ɣ
		**Cor**	39	-0.666	1.023	-0.446	0.860	-0.640	0.876	-0.546	1.188	0.780ɣ	0.16ɣ
		**Tran**	39	0.590	2.225	0.675	2.426	1.053	3.188	1.369	3.432	0.435ɣ	0.10ɣ
	**Ankle**	**Sag**	39	6.106	3.466	6.681	3.764	5.492	3.455	5.487	4.653	0.388ɣ	0.14ɣ
		**Cor**	39	0.792	4.305	0.609	5.112	1.536	5.054	1.856	7.674	0.557ɣ	0.09ɣ
		**Tran**	39	-2.509	5.009	-2.705	7.218	-0.862	4.951	-1.320	6.110	0.186ɣ	0.22*
**Event 2**	**Hip**	**Sag**	39	60.583	10.734	60.691	12.047	59.698	12.896	61.356	15.616	0.967*	0.01*
		**Cor**	39	-2.270	4.332	-3.618	6.381	-1.858	5.924	-1.676	10.045	0.719ɣ	0.06ɣ
		**Tran**	39	-3.101	7.098	-3.475	8.329	-4.447	7.894	-2.655	11.234	0.324ɣ	0.16ɣ
	**Knee**	**Sag**	39	50.445	13.224	51.701	12.690	53.883	10.226	53.830	11.426	0.195ɣ	0.21ɣ
		**Cor**	39	-1.445	5.424	-1.522	4.984	0.146	5.811	-0.205	8.897	0.136ɣ	0.24ɣ
		**Tran**	39	0.347	3.449	0.035	3.414	0.612	3.339	0.010	5.848	0.684ɣ	0.07ɣ
	**Ankle**	**Sag**	39	1.787	6.078	0.850	7.387	6.344	7.071	7.410	8.421	**0.004***	0.46*
		**Cor**	39	-1.187	4.801	-0.337	6.921	-1.407	5.780	-1.224	6.584	0.825ɣ	0.04ɣ
		**Tran**	39	-1.422	5.563	-2.361	7.874	-0.041	5.324	0.624	7.310	0.174ɣ	0.22ɣ
**Event 3**	**Hip**	**Sag**	39	23.381	13.365	21.085	12.480	20.069	8.765	19.624	10.472	0.264*	0.18*
		**Cor**	39	-4.917	2.696	-5.034	3.663	-3.420	2.303	-3.013	2.737	**0.020ɣ**	0.39ɣ
		**Tran**	39	-1.364	4.549	-1.507	4.575	-2.336	5.491	-1.818	7.195	0.275ɣ	0.18ɣ
	**Knee**	**Sag**	39	17.663	13.278	14.538	11.006	14.398	8.109	13.832	10.256	0.675*	0.07*

		**Cor**	39	-0.413	1.774	-0.407	1.751	-0.229	1.617	-0.423	1.880	0.512*	0.11*
		**Tran**	39	3.103	2.597	3.213	3.539	3.811	3.760	3.469	4.201	0.635*	0.08*
	**Ankle**	**Sag**	39	3.727	3.884	3.290	4.372	3.589	3.095	3.162	3.838	0.845γ	0.03γ
		**Cor**	39	4.666	5.296	3.861	6.652	3.475	4.110	4.178	6.425	0.229γ	0.20γ
		**Tran**	39	-1.778	4.902	-1.443	7.176	0.942	5.489	0.370	6.463	**0.024γ**	0.38γ

γ = paired-samples t-test; * = Wilcoxon signed-rank test; d= Cohen's d; r= rank–biserial correlation.

Table S4. Trailing-leg descriptive statistics and paired-samples t-test/Wilcoxon significance testing for triplanar (sagittal, coronal, transverse) lower-limb joint angles (hip, knee, ankle) across step-up events 1-3 (Event 1: initial stance; Event 2: box contact; Event 3: ascent completion).

Event	Joint	Plane	N	Affected Mean (º)	Affected SD (º)	Affected Median (º)	Affected IQR (º)	Contralateral Mean (º)	Contralateral SD (º)	Contralateral Median (º)	Contralateral IQR (º)	p	d/r
**Event 1**	**Hip**	**Sag**	39	20.008	9.478	19.672	11.888	23.069	8.805	21.752	11.799	**0.018***	0.22*
		**Cor**	39	-0.928	3.575	-0.928	5.897	-1.403	2.972	-1.174	4.141	0.566$^{\gamma}$	0.07$^{\gamma}$
		**Tran**	39	-2.763	6.358	-0.767	10.085	-2.056	5.195	-2.073	8.477	0.555$^{\gamma}$	0.40$^{\gamma}$
	**Knee**	**Sag**	39	18.501	8.446	17.154	10.820	20.828	9.143	21.372	13.224	0.176$^{\gamma}$	0.09$^{\gamma}$
		**Cor**	39	-1.123	1.642	-0.708	2.028	-1.038	2.462	-0.660	1.777	0.665$^{\gamma}$	0.10$^{\gamma}$
		**Tran**	39	-1.436	2.922	-0.784	4.034	-0.959	3.735	-0.869	4.787	0.449$^{\gamma}$	0.12$^{\gamma}$
	**Ankle**	**Sag**	39	5.394	3.694	5.450	6.285	6.295	3.883	5.858	5.701	0.209$^{\gamma}$	0.20$^{\gamma}$
		**Cor**	39	-2.143	4.273	-1.578	6.108	-2.617	5.013	-2.705	7.501	0.693$^{\gamma}$	0.06$^{\gamma}$
		**Tran**	39	-0.721	5.398	-0.599	10.193	-2.489	5.571	-2.559	7.424	0.176$^{\gamma}$	0.22$^{\gamma}$
**Event 2**	**Hip**	**Sag**	39	9.738	10.430	8.501	12.059	9.067	9.795	6.441	9.859	0.477*	0.11*
		**Cor**	39	2.440	4.017	2.138	4.954	1.119	3.393	1.075	4.816	0.171$^{\gamma}$	0.22$^{\gamma}$
		**Tran**	39	-4.224	5.722	-3.500	6.316	-4.142	4.846	-3.805	5.597	0.937$^{\gamma}$	0.01$^{\gamma}$
	**Knee**	**Sag**	39	15.337	9.935	13.083	11.665	13.043	11.354	11.814	8.814	0.024*	0.36*
		**Cor**	39	-0.913	1.246	-1.027	1.570	-0.964	1.418	-0.990	0.910	0.686*	0.06*
		**Tran**	39	1.372	2.372	1.821	3.535	0.892	2.620	0.809	3.439	0.287$^{\gamma}$	0.17$^{\gamma}$
	**Ankle**	**Sag**	39	10.892	6.692	10.447	6.782	7.120	8.196	9.289	12.103	0.018*	0.38*
		**Cor**	39	1.998	4.135	2.695	4.388	2.368	4.715	2.238	6.160	0.802*	0.04*
		**Tran**	39	-0.220	4.438	-0.457	5.836	-1.532	6.422	-1.466	6.421	0.135*	0.24*
**Event 3**	**Hip**	**Sag**	39	23.694	10.252	24.189	13.092	23.450	8.944	22.604	11.325	0.645*	0.07*
		**Cor**	39	-1.294	2.821	-1.340	3.610	-2.704	2.341	-2.798	3.196	0.023$^{\gamma}$	0.38$^{\gamma}$
		**Tran**	39	-2.372	5.580	-1.388	7.243	-1.981	5.480	-3.050	5.351	0.683$^{\gamma}$	0.07$^{\gamma}$
	**Knee**	**Sag**	39	16.907	6.496	16.053	7.594	16.312	8.039	14.990	9.268	0.577$^{\gamma}$	0.23$^{\gamma}$
		**Cor**	39	1.461	1.716	1.244	2.142	0.620	1.880	0.533	2.139	0.015*	0.09*
		**Tran**	39	4.071	3.904	4.172	6.152	3.212	3.427	3.253	4.024	0.154*	0.39*
	**Ankle**	**Sag**	39	5.216	4.158	5.533	5.137	5.811	3.914	6.211	5.280	0.505$^{\gamma}$	0.11$^{\gamma}$
		**Cor**	39	1.813	4.119	2.730	5.692	4.196	4.151	4.100	5.885	0.017$^{\gamma}$	0.40$^{\gamma}$
		**Tran**	39	0.170	4.408	0.256	5.136	-2.020	4.406	-0.965	6.540	0.015$^{\gamma}$	0.41$^{\gamma}$

$^{\gamma}$ = paired-samples t-test; * = Wilcoxon signed-rank test; d= Cohen's d; r= rank–biserial correlation.

3. Step-Down (when step-up leading leg was affected)

3.1. EMG

3.1.1 Descriptive Statistics

Table S5. Leading-leg descriptive statistics and Mann–Whitney U significance testing for muscle activation (VM, VL, BF, ST) across step-down (when step-up leading leg was affected) events 4-6 (Event 4: descent initiation; Event 5: leading-leg touchdown; Event 6: descent completion).

Event	Muscle	Affected N	Affected Mean (%MVC)	Affected SD (%MVC)	Affected Median (%MVC)	Affected IQR (%MVC)	Contralateral N	Contralateral Mean (%MVC)	Contralateral SD (%MVC)	Contralateral Median (%MVC)	Contralateral IQR (%MVC)	p	r
Event 4	**VM**	35	15.3518	12.2082	12.0036	11.2618	15	13.6418	12.1032	9.8976	20.7259	0.385	0.12
	**VL**	35	14.2601	9.3121	12.2735	11.2609	15	10.7218	7.7037	12.0351	15.1379	0.374	0.13
	**BF**	35	11.5902	10.4375	8.5596	10.5877	15	8.7325	4.8520	9.0319	7.1360	0.865	0.02
	**ST**	35	6.8766	5.5697	5.0495	6.5191	15	7.4230	9.2183	3.1869	5.7342	0.385	0.12
Event 5	**VM**	35	10.9501	8.8956	8.3305	11.7991	15	10.5691	8.7440	9.8831	14.9811	0.816	0.03
	**VL**	35	10.7349	9.1300	7.1680	8.9338	15	8.9400	7.9506	6.0275	10.2618	0.539	0.09
	**BF**	35	6.1469	5.3566	4.1906	6.5124	15	6.7502	6.0724	4.1622	4.9949	0.832	0.03
	**ST**	35	4.1715	4.0696	2.7026	4.6458	15	5.3078	5.1252	3.7399	5.4111	0.352	0.13
Event 6	**VM**	35	6.1309	7.3930	3.6402	4.5973	15	6.2453	4.2258	7.6188	8.3325	0.498	0.10
	**VL**	35	5.0080	6.9514	2.9984	4.2176	15	5.1357	4.8040	4.1202	5.0712	0.582	0.08
	**BF**	35	5.7514	6.0055	3.1328	4.0383	15	4.3261	3.7362	2.4378	4.8702	0.330	0.14
	**ST**	35	6.4190	5.6330	4.3728	5.4322	15	2.8141	2.9032	2.0538	2.1189	0.004	0.40

Table S6. Trailing-leg descriptive statistics and Mann–Whitney U significance testing for muscle activation (VM, VL, BF, ST) across step-down (when step-up leading leg was affected) events 4-6 (Event 4: descent initiation; Event 5: leading-leg touchdown; Event 6: descent completion).

Event	Muscle	Affected N	Affected Mean (%MVC)	Affected SD (%MVC)	Affected Median (%MVC)	Affected IQR (%MVC)	Contralateral N (%MVC)	Contralateral Mean (%MVC)	Contralateral SD (%MVC)	Contralateral Median (%MVC)	Contralateral IQR (%MVC)	p	r
**Event 4**	VM	15	9.9377	5.1374	9.0896	8.2322	35	8.1917	4.5461	7.7732	6.1888	0.253	0.16
	VL	15	9.7512	3.7198	8.1312	7.4218	35	8.1262	5.0163	7.0306	4.5977	0.150	0.20
	BF	15	10.7147	8.5665	8.4696	8.5545	35	11.2293	14.7517	6.7562	7.2873	0.512	0.09
	ST	15	11.4006	7.7996	5.9752	14.1245	35	12.5609	11.9612	10.5013	11.7518	0.899	0.02
**Event 5**	VM	15	18.1039	12.5935	15.4298	16.3972	35	20.1853	12.6745	19.2510	13.9982	0.539	0.09
	VL	15	15.2826	9.0462	17.8115	15.0972	35	16.6004	8.3700	15.2080	9.7932	0.767	0.04
	BF	15	16.1579	11.8476	12.9071	14.5342	35	9.4663	13.5220	3.9927	12.2562	0.006	0.39
	ST	15	8.2240	3.4467	8.6501	5.7665	35	7.4139	7.4335	5.3335	6.0032	0.086	0.24
**Event 6**	VM	15	18.2886	8.3610	17.9066	13.3254	35	11.7260	12.0585	9.6035	8.9298	0.003	0.42
	VL	15	21.1288	14.8666	16.3634	7.9259	35	10.1579	4.4645	9.9501	5.0818	0.000	0.57
	BF	15	18.6797	13.3678	13.1593	14.0522	35	12.3821	15.5469	5.6891	9.8687	0.009	0.37
	ST	15	12.8899	12.1995	10.1951	8.5160	35	9.9253	7.8729	6.3335	11.9158	0.472	0.10

3.2. Kinematics

3.2.1 Descriptive Statistics

Table S7. Leading-leg descriptive statistics and Mann–Whitney U significance testing for triplanar (sagittal, coronal, transverse) lower-limb joint angles (hip, knee, ankle) across step-down (when step-up leading-leg was affected) events 4-6 (Event 4: descent initiation; Event 5: leading leg touchdown; Event 6: descent completion).

Event	Joint	Plane	Affected N	Affected Mean (º)	Affected SD (º)	Affected Median (º)	Affected IQR (º)	Contralateral N	Contralateral Mean (º)	Contralateral SD (º)	Contralateral Median (º)	Contralateral IQR (º)	p	r
**Event 4**	Hip	Sag	35	15.1152	8.8511	15.6045	10.1333	15	17.4958	9.1734	17.0334	11.1961	0.966	0.01
		Cor	35	-2.5492	2.7121	-2.9251	1.8148	15	-3.3684	1.9813	-3.3446	1.9842	0.000	0.53
		Tran	35	-2.4775	5.2023	-4.1532	7.7621	15	-2.1816	4.7346	-1.9907	6.6511	0.485	0.10
	Knee	Sag	35	12.0125	9.8615	9.7879	8.1632	15	12.9932	6.9824	11.9002	7.4495	0.446	0.11
		Cor	35	-0.7427	2.3148	-0.1982	1.4070	15	-0.0625	1.2023	-0.0029	1.4944	0.253	0.16
		Tran	35	2.7051	3.9446	2.5245	4.8747	15	3.6490	2.7827	3.0633	4.0312	0.703	0.05
	Ankle	Sag	35	7.0951	3.4578	6.5198	5.0337	15	5.4476	3.3744	6.5619	4.3570	0.066	0.26
		Cor	35	4.2863	4.2883	3.8902	5.9263	15	4.0702	3.3478	4.7349	4.1322	0.103	0.23
		Tran	35	-0.6307	3.6991	0.5219	4.9096	15	0.4093	5.2373	0.1552	6.7295	0.320	0.14
**Event 5**	Hip	Sag	35	21.6962	9.7783	23.8014	12.7172	15	22.8911	11.8601	23.5876	18.6543	0.735	0.05
		Cor	35	3.3357	4.4981	4.5152	5.6874	15	3.0487	5.6001	1.4705	5.3573	0.582	0.08
		Tran	35	-7.0050	5.5179	-8.8543	7.3626	15	-7.0055	7.3875	-4.9922	10.9495	0.099	0.23
	Knee	Sag	35	14.1629	14.6731	7.0973	15.8542	15	13.2959	13.6520	10.0385	10.8016	0.512	0.09
		Cor	35	0.8525	1.5444	0.9400	2.2686	15	0.5954	2.2539	0.4404	2.0805	0.112	0.22
		Tran	35	2.0848	4.1098	2.1256	6.5807	15	-0.3157	4.3367	-0.4877	6.6194	0.767	0.04
	Ankle	Sag	35	-7.4835	15.2994	-7.9643	26.9739	15	-14.9337	19.6874	-23.7971	35.4695	0.866	0.02
		Cor	35	-4.5201	8.5899	-2.9337	13.5137	15	-6.2186	7.7920	-8.3048	11.6355	0.582	0.08
		Tran	35	0.9221	6.5203	0.9447	8.9440	15	4.3634	6.3899	5.9188	9.4359	0.079	0.25
**Event 6**	Hip	Sag	35	2.8654	11.4840	3.7728	18.1703	15	2.7483	10.8053	1.4708	17.3862	0.703	0.05
		Cor	35	-1.7389	2.7042	-0.5517	2.4245	15	-0.6698	4.0708	-1.4111	4.7002	0.138	0.21
		Tran	35	0.0865	4.9383	-0.0624	6.1793	15	1.9074	8.7556	3.5932	9.4517	0.127	0.22
	Knee	Sag	35	17.1905	15.5364	13.2160	13.0863	15	19.0039	15.7281	17.8428	10.2906	0.816	0.03
		Cor	35	-2.0668	1.4919	-2.1285	2.7995	15	-1.2002	1.7438	-1.0448	2.2841	0.122	0.22
		Tran	35	-2.0622	3.8584	-0.5498	5.4870	15	0.5077	4.1991	1.0421	5.5377	0.300	0.15
	Ankle	Sag	35	13.2347	6.8930	14.6944	8.6442	15	14.1752	6.9567	15.7201	6.2476	0.949	0.01
		Cor	35	-0.6777	4.7919	-0.2001	7.3121	15	1.3032	5.3982	2.4333	6.2567	0.611	0.07
		Tran	35	0.8373	5.1009	1.2165	6.2568	15	1.8305	5.8264	2.1632	8.1730	0.799	0.04

Table S8. Trailing-leg descriptive statistics and Mann–Whitney U significance testing for triplanar (sagittal, coronal, transverse) lower-limb joint angles (hip, knee, ankle) across step-down (when step-up leading leg was affected) events 4-6 (Event 4: descent initiation; Event 5: leading-leg touchdown; Event 6: descent completion).

Event	Joint	Plane	Affected N	Affected Mean (°)	Affected SD (°)	Affected Median (°)	Affected IQR (°)	Contralateral N	Contralateral Mean (°)	Contralateral SD (°)	Contralateral Median (°)	Contralateral IQR (°)	p	r
**Event 4**	Hip	Sag	15	21.6424	9.0424	22.8887	10.4786	35	20.9699	10.0450	23.1962	14.0712	0.539	0.09
		Cor	15	-3.0049	2.0717	-3.2626	2.6497	35	-2.7498	2.9512	-2.5312	2.2479	0.012	0.36
		Tran	15	-2.0055	4.1667	-2.4115	4.7627	35	-2.2934	6.6493	-1.5362	4.9672	0.899	0.02
	Knee	Sag	15	14.6937	7.7042	14.1616	8.8823	35	15.6565	8.6818	12.8605	10.4198	0.611	0.07
		Cor	15	0.4102	1.2761	0.3771	1.5244	35	0.2263	3.0481	-0.4477	2.0507	0.103	0.23
		Tran	15	2.5574	2.9988	2.1908	3.0777	35	3.2319	5.3768	2.3816	4.3097	0.374	0.13
	Ankle	Sag	15	4.7460	3.3369	5.5194	4.5960	35	6.8462	4.0793	7.0805	3.8347	0.597	0.07
		Cor	15	3.7705	4.4756	2.8929	5.3417	35	2.8685	4.5351	3.9882	5.3843	0.138	0.21
		Tran	15	-2.6568	4.7164	-1.8588	6.3368	35	1.2751	5.4235	0.6307	5.4381	0.212	0.18
**Event 5**	Hip	Sag	15	17.1804	13.2651	17.0208	16.2091	35	22.1310	13.2166	23.1255	17.1610	0.341	0.13
		Cor	15	-5.6693	5.9397	-5.8087	8.3501	35	-5.6625	4.3424	-6.3558	4.0395	0.916	0.01
		Tran	15	3.3445	6.5836	2.7417	9.9877	35	2.6891	6.4596	4.7003	7.5602	0.553	0.08
	Knee	Sag	15	44.0166	19.4839	42.5581	18.0130	35	52.4706	19.5951	50.7496	23.8233	0.156	0.20
		Cor	15	-1.3954	4.0609	-1.5709	4.8451	35	0.7190	4.6687	-0.2656	6.3925	0.525	0.09
		Tran	15	0.9766	3.1833	1.0842	4.6657	35	1.5996	3.8136	2.0909	5.9919	0.783	0.04
	Ankle	Sag	15	19.7521	12.5166	22.0899	17.8040	35	20.7959	10.4808	19.4324	16.9571	0.086	0.24
		Cor	15	4.3355	6.7583	4.6751	7.9035	35	3.3445	6.2269	1.8615	9.6914	0.374	0.13
		Tran	15	-2.5573	6.1792	-3.1904	9.9666	35	1.1721	5.5279	2.2295	6.6492	0.799	0.04
**Event 6**	Hip	Sag	15	25.0398	10.6347	25.4565	12.5826	35	23.3830	11.7532	24.7733	10.6632	0.409	0.12
		Cor	15	-0.6495	5.1619	-1.3954	7.3946	35	-3.7668	5.9434	-5.6275	8.7847	0.719	0.05
		Tran	15	-3.0134	7.3146	-2.8375	6.2550	35	-5.9645	7.1857	-6.0418	11.9806	0.641	0.07
	Knee	Sag	15	10.1992	8.0319	8.9621	8.2554	35	8.7818	9.6335	5.5745	9.9680	0.498	0.10
		Cor	15	0.8495	1.7499	0.9207	1.4880	35	-0.0505	1.3028	-0.0603	1.9614	0.290	0.15
		Tran	15	1.9232	5.1613	2.6264	6.3534	35	-0.5773	4.8242	0.0292	5.2377	0.626	0.07
	Ankle	Sag	15	-2.4358	7.3541	-3.2097	8.9172	35	-1.7692	8.8275	-0.8561	8.1155	0.409	0.12
		Cor	15	-4.6120	7.2511	-4.8318	8.0839	35	0.4271	7.0953	2.0388	9.9282	0.688	0.06
		Tran	15	-0.7657	5.4059	-0.2568	7.3706	35	-0.0287	4.8681	1.0581	9.0093	0.949	0.01

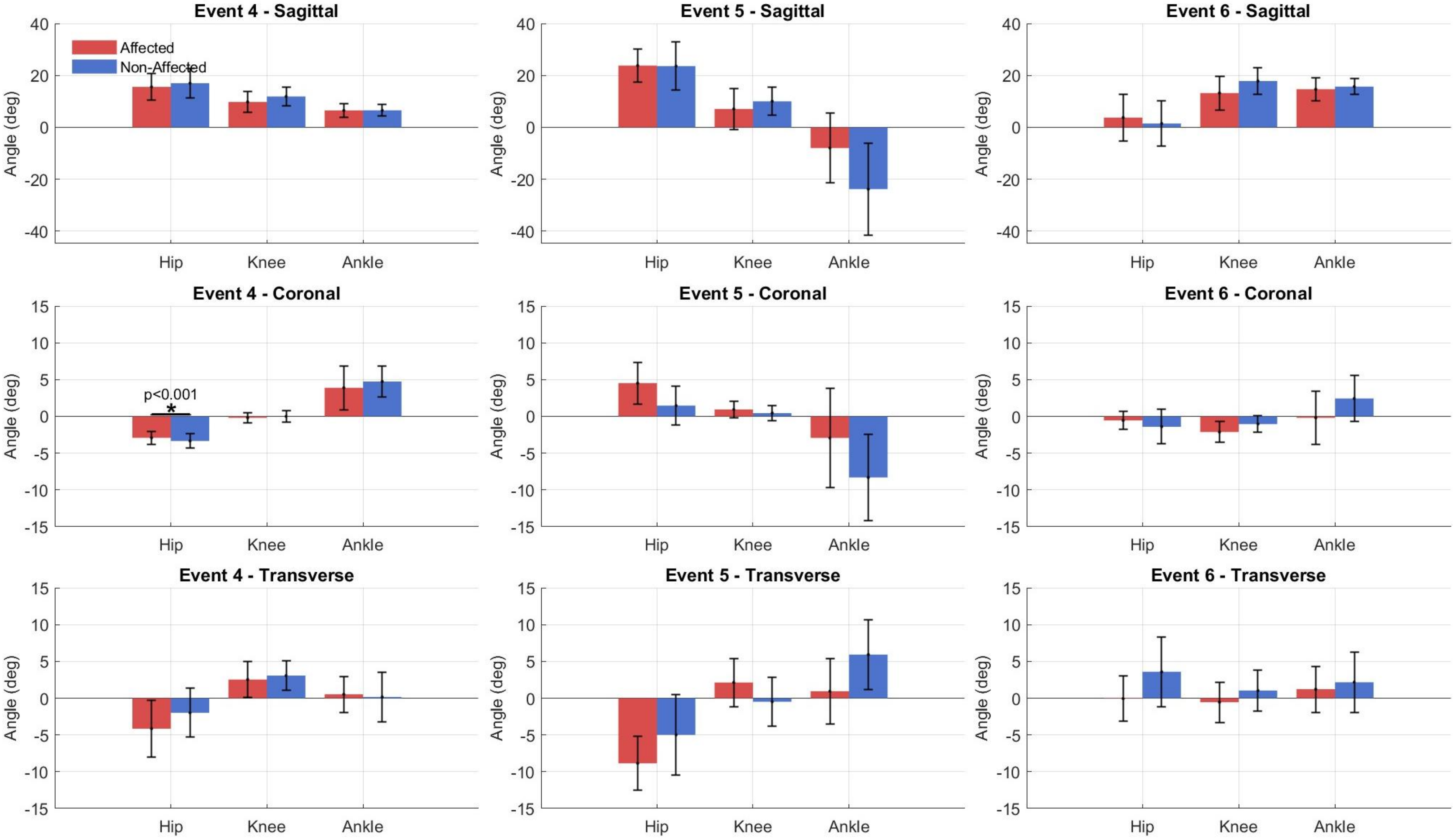

Figure S2. Comparison of hip, knee, and ankle joint angles in <u>leading limbs</u> between affected (red) and contralateral (blue) sides during a) step-down (when step-up leading limb was affected) events; b) step-down (when step-up leading limb was <u>contralateral</u>) events. Event 4: descend initiation; Event 5: leading-leg touchdown; Event 6: descent completion. Significant differences ($p < 0.05$) were observed between affected and contralateral limbs across Events 4-6.

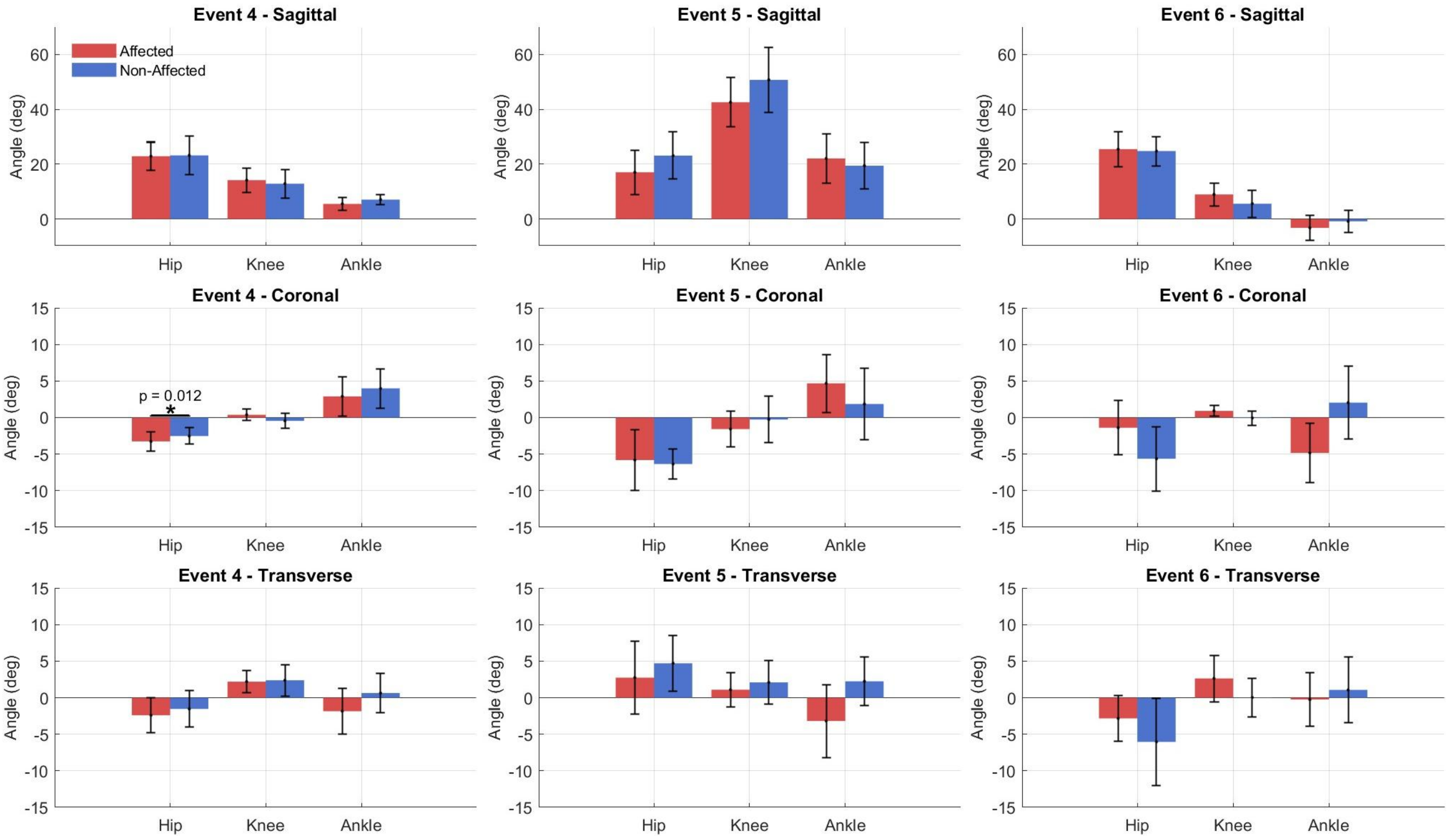

Figure S3. Comparison of hip, knee, and ankle joint angles in trailing limbs between affected (red) and contralateral (blue) sides during a) step-down (when step-up leading limb was affected) events; b) step-down (when step-up leading limb was contralateral) events. Event 4: descent initiation; Event 5: leading leg touchdown; Event 6: descent completion. Significant differences ($p < 0.05$) were observed between affected and contralateral limbs across Events 4-6.

4. Step-Down (when step-up leading leg was contralateral)

4.1. EMG

4.1.1 Descriptive Statistics

Table S9. Leading-leg descriptive statistics and Mann–Whitney U significance testing for muscle activation (VM, VL, BF, ST) across step-down (when step-up leading leg was contralateral) events 4-6 (Event 4: descent initiation; Event 5: leading-leg touchdown; Event 6: descent completion).

Event	Muscle	Affected N	Affected Mean (%MVC)	Affected SD (%MVC)	Affected Median (%MVC)	Affected IQR (%MVC)	Contralateral N	Contralateral Mean (%MVC)	Contralateral SD (%MVC)	Contralateral Median (%MVC)	Contralateral IQR	p	r
Event 4	VM	17	13.9067	20.0992	9.2197	10.1195	34	10.9089	7.2227	10.3064	8.5988	0.639	0.07
	VL	17	9.0283	8.5114	6.9368	5.7196	34	9.7872	6.1219	8.6559	7.6023	0.374	0.12
	BF	17	9.5048	8.3481	6.3405	10.2991	34	8.2102	8.4300	5.3171	6.0291	0.569	0.08
	ST	17	9.3092	6.0358	9.5135	7.2502	34	5.3306	4.0335	4.0057	5.8153	0.014	0.34
Event 5	VM	17	13.3765	13.2801	11.2029	14.3622	34	11.7687	7.8092	11.3354	11.5945	0.881	0.02
	VL	17	10.3870	8.3593	7.1669	10.5132	34	10.2627	11.4594	6.9544	10.7637	0.757	0.04
	BF	17	11.5724	16.0186	6.7499	11.2602	34	7.6198	8.0724	4.3097	10.4430	0.529	0.09
	ST	17	8.2758	6.5987	7.0970	7.6378	34	5.8503	6.8597	3.4113	6.4641	0.080	0.24
Event 6	VM	17	6.9206	6.5776	4.6971	7.9753	34	8.4375	6.5642	7.6214	7.6917	0.503	0.09
	VL	17	6.5899	7.7274	2.9097	7.4905	34	7.2711	5.9553	6.0892	6.9478	0.303	0.14
	BF	17	5.5155	4.9315	3.9720	7.0184	34	4.9540	5.5019	3.4292	3.3682	0.803	0.03
	ST	17	5.4659	5.1716	3.1653	6.5717	34	3.1163	2.8868	2.2991	2.5144	0.147	0.20

Table S10. Trailing-leg descriptive statistics and Mann–Whitney U significance testing for muscle activation (VM, VL, BF, ST) across step-down (when step-up leading-leg was contralateral) events 4-6 (Event 4: descent initiation; Event 5: leading-leg touchdown; Event 6: descent completion).

Event	Muscle	Affected N	Affected Mean (%MVC)	Affected SD (%MVC)	Affected Median (%MVC)	Affected IQR (%MVC)	Contralateral N	Contralateral Mean (%MVC)	Contralateral SD (%MVC)	Contralateral Median (%MVC)	Contralateral IQR (%MVC)	p	r
Event 4	**VM**	15	14.0002	9.1353	14.3488	11.1396	35	5.8199	5.1461	4.0026	3.7798	0.000	0.50
	**VL**	15	12.6566	6.5312	11.3625	9.2684	35	8.2365	6.3848	6.5947	8.6145	0.015	0.34
	**BF**	15	12.4571	7.4722	10.2304	9.7047	35	7.5341	5.6504	6.6585	6.1549	0.007	0.38
	**ST**	15	13.6803	11.3487	10.6113	13.3744	35	7.5782	3.6125	8.0925	4.3022	0.084	0.24
**Event**	**VM**	15	20.1125	14.9273	17.3074	17.2459	35	17.7595	15.3069	14.5829	22.5371	0.610	0.07

**5**	**VL**	15	17.9884	15.6080	16.2495	13.7816	35	14.8946	10.2092	18.8295	21.0676	0.897	0.02
	**BF**	15	12.7812	9.1300	10.4551	9.8244	35	8.9886	14.3469	2.4708	7.5315	0.006	0.39
	**ST**	15	7.7126	6.7351	4.8429	8.9181	35	7.0445	8.0151	3.6662	7.6868	0.418	0.11
**Event 6**	**VM**	15	11.0408	6.8079	10.2268	10.8217	35	7.1399	3.8138	6.6266	6.5649	0.071	0.25
	**VL**	15	11.0216	7.4995	8.6420	5.5568	35	8.1138	5.0190	8.3835	6.8782	0.212	0.17
	**BF**	15	13.2662	8.7198	10.9359	12.4315	35	11.1887	8.0241	9.7871	11.6057	0.466	0.10
	**ST**	15	11.0624	8.0692	9.7653	9.7642	35	6.8726	5.6478	4.7280	2.7924	0.027	0.31

4.2. Kinematics

4.2.1 Descriptive Statistics

Table S11. Leading-leg descriptive statistics and Mann–Whitney U significance testing for triplanar (sagittal, coronal, transverse) lower-limb joint angles (hip, knee, ankle) across step-down (when step-up leading-leg was contralateral) events 4-6 (Event 4: descent initiation; Event 5: leading-leg touchdown; Event 6: descent completion).

Event	Joint	Plane	Affected N	Affected Mean (°)	Affected SD (°)	Affected Median (°)	Affected IQR (°)	Contralateral N	Contralateral Mean (°)	Contralateral SD (°)	Contralateral Median (°)	Contralateral IQR (°)	p	r
**Event 4**	Hip	Sag	34	14.9527	8.6145	15.4912	8.3450	18	17.3869	9.0556	16.2924	10.7829	0.977	0.00
		Cor	34	-2.5585	2.6314	-2.9208	1.6864	18	-3.5293	2.1651	-3.3580	1.9687	0.096	0.23
		Tran	34	-2.7545	5.1820	-4.3490	8.4352	18	-2.1230	4.6748	-1.9351	6.5060	0.519	0.09
	Knee	Sag	34	12.1977	9.5993	10.5814	8.1484	18	13.2371	7.0213	11.9583	7.6181	0.341	0.13
		Cor	34	-0.6366	2.2903	-0.0342	1.4007	18	-0.0848	1.1911	-0.0185	1.5168	0.403	0.12
		Tran	34	2.9471	3.9622	3.2237	5.0382	18	3.6733	2.7439	3.1090	3.9242	0.381	0.12
	Ankle	Sag	34	7.3107	3.4771	7.3977	4.6287	18	5.5328	3.3599	6.5870	4.2541	0.825	0.03
		Cor	34	4.1690	4.1899	3.4620	5.8465	18	4.1062	3.3034	4.8064	4.0298	0.303	0.14
		Tran	34	-0.9337	3.8120	0.1829	6.8490	18	0.1133	5.4384	-0.0924	6.8791	0.946	0.01
**Event 5**	Hip	Sag	34	22.5015	10.0827	23.8467	14.3825	18	23.3399	11.9686	25.4894	18.5546	0.403	0.12
		Cor	34	3.2071	4.3978	4.4976	5.2068	18	2.9550	5.5416	1.4172	5.2987	0.092	0.23
		Tran	34	-6.8975	5.3726	-7.9922	7.0952	18	-7.1394	7.3164	-5.0270	10.1108	0.096	0.23
	Knee	Sag	34	15.9058	16.0409	10.0666	16.7510	18	13.7886	13.7471	10.0648	10.7760	0.392	0.12
		Cor	34	1.0509	1.7185	1.0222	2.2658	18	0.5643	2.2268	0.3817	2.0608	0.780	0.04
		Tran	34	2.3340	4.1249	2.2866	6.7023	18	-0.2715	4.2782	0.0622	6.3312	0.795	0.04
	Ankle	Sag	34	-7.0655	14.9481	-3.9620	25.5131	18	-14.9136	19.3871	-23.5770	34.1396	0.361	0.13
		Cor	34	-4.6390	8.3487	-3.4009	12.2560	18	-6.4390	7.7798	-8.7193	11.8468	0.855	0.03
		Tran	34	0.3076	6.8418	0.4597	8.8705	18	4.1779	6.3846	5.0828	9.3923	0.037	0.29
**Event 6**	Hip	Sag	34	2.1636	11.5321	3.7566	18.4022	18	2.7107	10.6426	1.4708	17.0967	0.117	0.22
		Cor	34	-1.6476	2.6519	-0.4729	2.0018	18	-0.8104	4.0916	-1.4467	4.7399	0.693	0.05
		Tran	34	0.3435	4.9134	0.0605	7.7118	18	1.6749	8.7279	3.4199	9.6297	0.736	0.05
	Knee	Sag	34	16.7886	15.1687	12.6470	12.1745	18	18.6089	15.6583	17.8261	11.5676	0.870	0.02
		Cor	34	-2.1420	1.4821	-2.4897	2.8138	18	-1.1633	1.7306	-0.9710	2.3302	0.810	0.03
		Tran	34	-2.5373	4.2513	-0.8080	5.5697	18	0.5105	4.1350	0.9094	5.1257	0.252	0.16
	Ankle	Sag	34	13.5592	6.8275	14.7033	10.2977	18	14.1061	6.8623	15.5471	6.6026	0.041	0.28
		Cor	34	-1.0145	4.8634	-0.5801	7.6455	18	1.3799	5.3346	2.5096	6.0581	0.260	0.16
		Tran	34	0.8113	4.9498	0.7933	5.7956	18	1.6042	5.8874	1.5692	8.7952	0.019	0.32

Table S12. Trailing-leg descriptive statistics and Mann–Whitney U significance testing for triplanar (sagittal, coronal, transverse) lower-limb joint angles (hip, knee, ankle) across step-down (when step-up leading-leg was contralateral) events 4-6 (Event 4: descent initiation; Event 5: leading-leg touchdown; Event 6: descent completion).

Event	Joint	Plane	Affected N	Affected Mean (º)	Affected SD (º)	Affected Median (º)	Affected IQR (º)	Contralateral N	Contralateral Mean (º)	Contralateral SD (º)	Contralateral Median (º)	Contralateral IQR (º)	p	r
**Event 4**	Hip	Sag	18	21.6790	8.9069	22.8887	10.3820	34	20.4556	9.9864	21.1763	15.7489	0.679	0.06
		Cor	18	-2.9365	2.0787	-3.1868	2.9028	34	-2.8775	2.9139	-2.6926	2.7180	0.751	0.04
		Tran	18	-1.9685	4.1088	-2.3113	4.5806	34	-2.3627	6.4574	-2.2565	4.8023	0.736	0.05
	Knee	Sag	18	14.9406	7.7220	14.3328	9.2501	34	15.3345	8.5327	12.6638	9.5194	0.931	0.01
		Cor	18	0.4517	1.2797	0.4611	1.6840	34	0.2316	2.9572	-0.2860	1.8512	0.066	0.25
		Tran	18	2.5817	2.9565	2.5316	2.9599	34	3.3738	5.2509	2.4143	4.8993	0.751	0.04
	Ankle	Sag	18	4.8010	3.3016	5.7018	4.4451	34	6.8986	3.9637	7.1933	3.7162	0.049	0.27
		Cor	18	3.7209	4.4167	2.8854	5.1283	34	2.9536	4.4144	4.1500	4.4017	0.946	0.01
		Tran	18	-2.7932	4.7120	-2.2749	7.1239	34	0.6908	5.8164	0.5834	5.3741	0.028	0.31
**Event 5**	Hip	Sag	18	17.2809	13.0757	17.6658	15.6286	34	21.6536	12.9810	21.9358	15.9307	0.222	0.17
		Cor	18	-5.6270	5.8542	-5.6547	8.1945	34	-5.8344	4.2754	-6.6442	3.9293	0.901	0.02
		Tran	18	3.4262	6.5006	3.3027	9.8485	34	2.4164	6.3726	4.6913	8.5887	0.795	0.04
	Knee	Sag	18	43.4541	19.4647	41.8124	19.9681	34	50.9897	20.0213	49.8567	27.6377	0.194	0.18
		Cor	18	-1.3250	4.0200	-1.4870	4.9368	34	0.6648	4.5351	-0.2609	6.1637	0.146	0.20
		Tran	18	0.9965	3.1368	1.1780	4.3262	34	1.9197	3.9412	2.2054	6.5781	0.361	0.13
	Ankle	Sag	18	19.5470	12.3833	21.7279	17.3450	34	20.7201	10.1729	19.4324	16.2171	0.962	0.01
		Cor	18	4.3455	6.6554	4.6751	7.7019	34	3.6936	6.2199	3.1560	10.7109	0.795	0.04
		Tran	18	-2.7808	6.2228	-3.2984	9.9611	34	0.5038	6.0663	1.4973	7.6063	0.085	0.24
**Event 6**	Hip	Sag	18	24.9740	10.4793	25.2727	12.2110	34	23.0905	11.4696	24.6615	11.5959	0.597	0.07
		Cor	18	-0.7397	5.1102	-1.6370	7.1304	34	-4.1044	5.9411	-6.1462	8.3007	0.023	0.32
		Tran	18	-3.1049	7.2226	-2.9093	5.8263	34	-6.2978	7.1131	-6.4861	12.6282	0.126	0.21
	Knee	Sag	18	10.2672	7.9191	9.1342	7.9172	34	8.4745	9.4363	5.5423	9.3732	0.303	0.14
		Cor	18	0.9066	1.7549	0.9829	1.5230	34	0.0451	1.3273	0.1674	2.1216	0.039	0.29
		Tran	18	2.1714	5.2846	2.9416	6.7506	34	-0.5068	4.6897	0.0394	4.5956	0.053	0.27
	Ankle	Sag	18	-2.2331	7.3377	-2.4540	8.9431	34	-1.8401	8.5692	-1.5526	7.7068	0.825	0.03
		Cor	18	-4.7622	7.1939	-5.1378	7.7081	34	0.2756	6.9134	-0.0006	9.1872	0.010	0.36
		Tran	18	-0.9655	5.4494	-0.6483	7.3922	34	-0.4882	5.1092	1.0363	9.1252	0.532	0.09

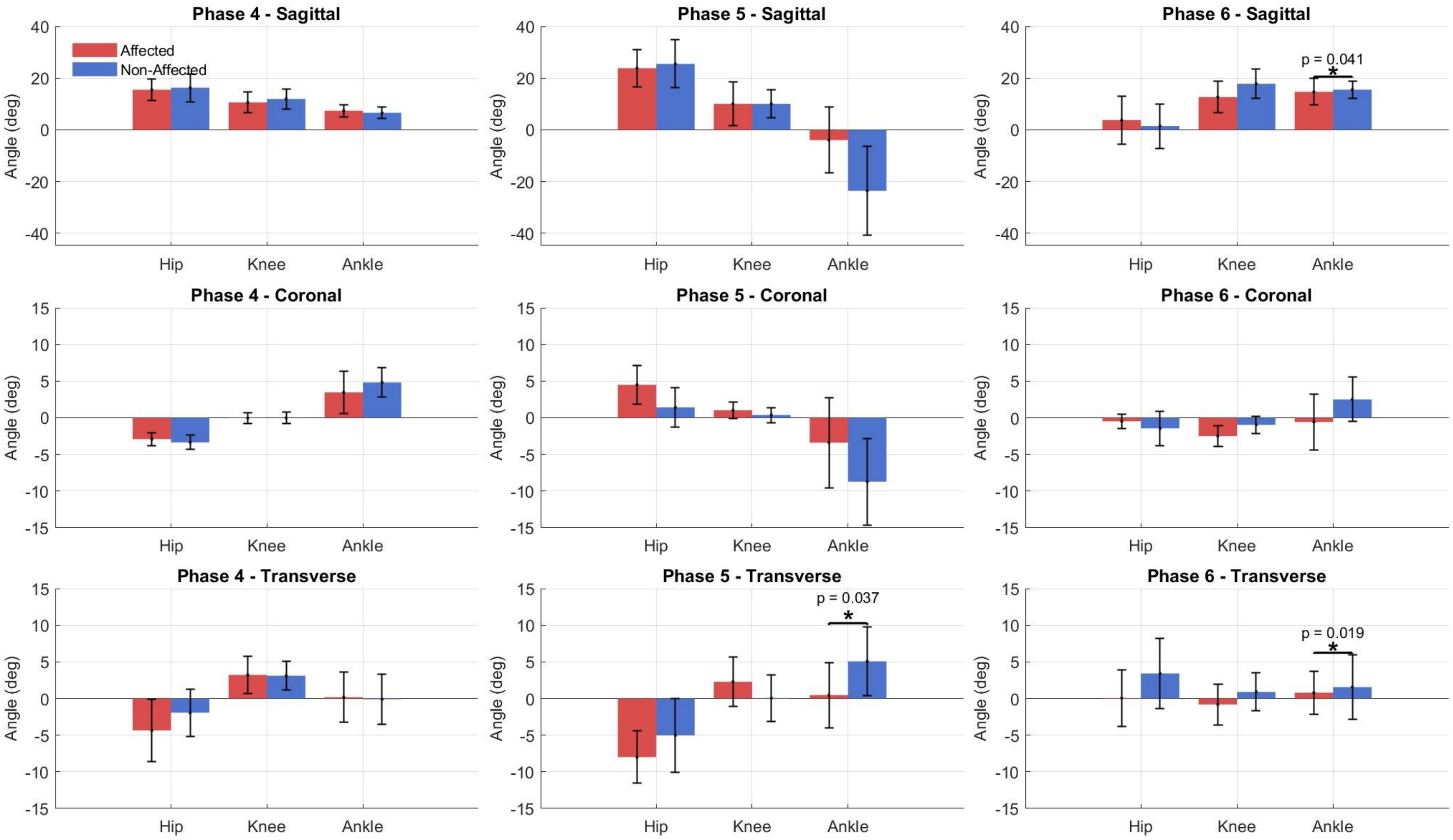

Figure S4 Comparison of hip, knee, and ankle joint angles in <u>leading limbs</u> between affected (red) and contralateral (blue) sides during a) step-down (when step-up leading limb was affected) events; b) step-down (when step-up leading limb was <u>contralateral</u>) events. Event 4: descent initiation; Event 5: leading-leg touchdown; Event 6: descent completion. Significant differences ($p < 0.05$) were observed between affected and contralateral limbs across Events 4-6.

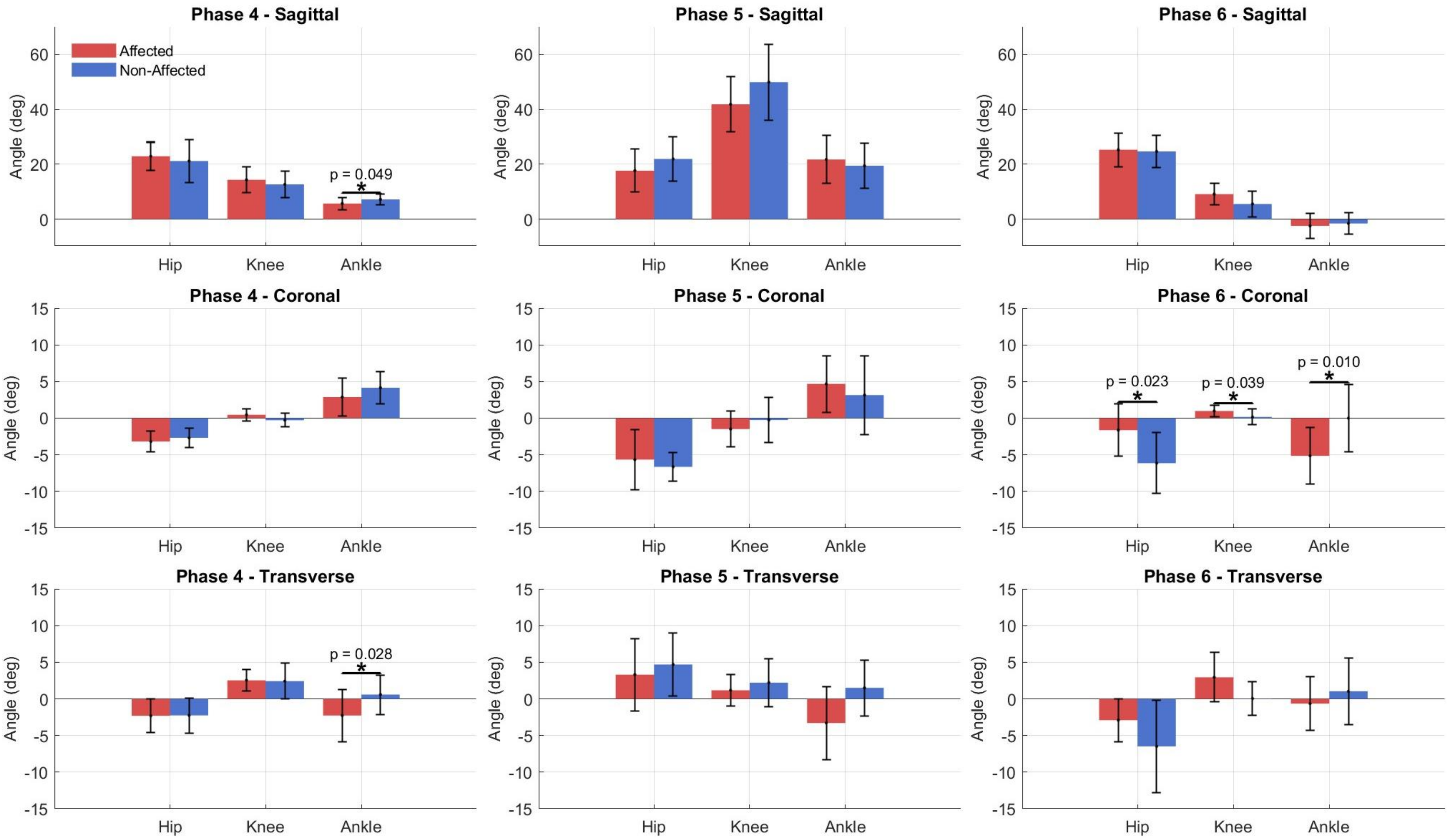

Figure S5. Comparison of hip, knee, and ankle joint angles in trailing limbs between affected (red) and contralateral (blue) sides during a) step-down (when step-up leading limb was affected) events; b) step-down (when step-up leading limb was contralateral) events. Event 4: descent initiation; Event 5: leading-leg touchdown; Event 6: descent completion. Significant differences ($p < 0.05$) were observed between affected and contralateral limbs across Events 4-6.

1. Load Data

Load affected leg info, synchronized IMU, force platform, and joint angle data

Initialize arrays for segment positions, accelerations, and knee angles

2. Extract signals:

For each subject and session:

For trials of interest:

Extract Left and Right Foot Z-axis positions

Extract Left and Right Foot Z-axis accelerations

Extract Left and Right Knee flexion/extension angles

3. Event Detection for Dominant Leg

For each subject and session:

Extract affected leg (R or L)

Assign step-up leading and trailing knee angles and trailing leg position

Extract Platform 1 and Platform 2 force data

3.1. Event 2: Box Load Start

Compute difference signal (PL1diff)

Find peaks in PL1diff with MinPeakHeight ≥ 0

Remove peaks closer than 200 frames

For each peak:

Find last negative-to-positive transition before peak

Store transition frame

Save all transitions as Event 2 frames (ignore frames < 5)

3.2. Event 1:

Find knee extension peaks in dominant knee angle.

For each Event 2 frame: Identify all peaks before box load frame

If ≥ 2 peaks: select last peak.

If 1 peak: select that peak.

If none: mark NaN.

Store selected peak frame as Event 1.

3.3. Event 3:

For each Event 2 frame:

Define window: startFrame = Event 2 frame + 50, endFrame = startFrame + 200.

Search Platform 1 vertical GRF differential for first negative-to-positive transition in window

If found: store as box load end frame

For each cycle:

Extract trailing leg position between startFrame and box load end frame

Find first peak (≥ 0.25)

After peak, find first valley

Compute final frame = startFrame + valley offset

Else: mark NaN

Store as Event 3

3.4. Event 5:

Compute difference signal (Pl2diff)

Find peaks in Pl2diff (MinPeakHeight ≥ 20)

For each peak:

Find last negative-to-positive transition before peak

Store transitions as Event 5 frames

3.5. Event 6:

For each Event 5 frame:

Determine step-down foot by comparing foot positions

For trailing foot:

Extract inverted position signal in window (startFrame +50 to +100)

Find first peak (MinPeakHeight ≤ -1)

Compute final frame = startFrame + peak offset

Store as Event 6

Refine timing:

Around Event 6 frame (−50 to +50):

Extract trailing foot z-axis acceleration

Find positive peaks > +5

Select closest peak to Phase 6 frame

Store refined frame as Event 6 Acc

3.6. Event 4:

For each Phase 5 frame:

Select step-down foot position

Extract data before step-down frame

Find valleys in inverted signal

Select last valley before step-down

If none: mark NaN

Store as Event 4

4. Save results

Save Event1– Event6, Event6Acc, StepDownFoot

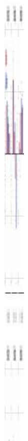

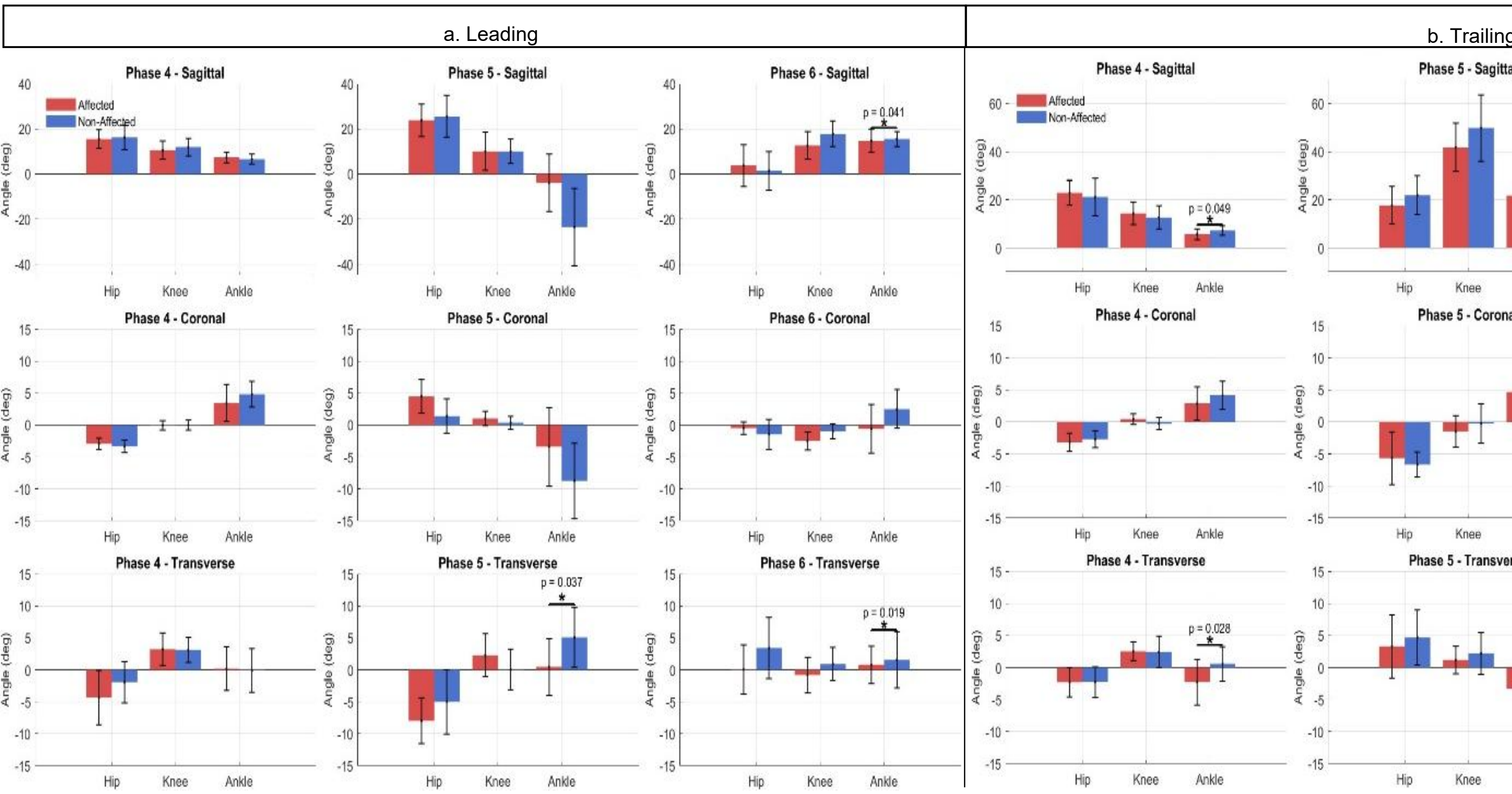

Figure x. Comparison of hip, knee, and ankle joint angles for leading (a) and trailing limbs (b) between affected (red) and contralateral (blue) s leading limb was contralateral) cycle. Solid red line = affected limb; solid blue line = contralateral limb. Event 4 (descend initiation) occurs at 0%. Ev by dotted lines (red = affected, blue = contralateral). Event 6 (descend completion) occurs at 100%.
